# Differential intracellular management of fatty acids impacts on metabolic stress-stimulated glucose uptake in cardiomyocytes

**DOI:** 10.1038/s41598-023-42072-7

**Published:** 2023-09-08

**Authors:** Ettore Vanni, Karina Lindner, Anne-Claude Gavin, Christophe Montessuit

**Affiliations:** 1 Department of Pathology and Immunology, University of Geneva School of Medicine, Geneva, Switzerland; 2 Department of Cell Physiology and Metabolism, University of Geneva School of Medicine, Geneva, Switzerland

**Keywords:** Lipidomics, Stress signalling, Diabetes, Metabolism, Fat metabolism

## Abstract

Stimulation of glucose uptake in response to ischemic metabolic stress is important for cardiomyocyte function and survival. Chronic exposure of cardiomyocytes to fatty acids (FA) impairs the stimulation of glucose uptake, whereas induction of lipid droplets (LD) is associated with preserved glucose uptake. However, the mechanisms by which LD induction prevents glucose uptake impairment remain elusive. We induced LD with either tetradecanoyl phorbol acetate (TPA) or 5-aminoimidazole-4-carboxamide-1-β-D-ribofuranoside (AICAR). Triacylglycerol biosynthesis enzymes were inhibited in cardiomyocytes exposed to FA ± LD inducers, either upstream (glycerol-3-phosphate acyltransferases; GPAT) or downstream (diacylglycerol acyltransferases; DGAT) of the diacylglycerol step. Although both inhibitions reduced LD formation in cardiomyocytes treated with FA and LD inducers, only DGAT inhibition impaired metabolic stress-stimulated glucose uptake. DGAT inhibition in FA plus TPA-treated cardiomyocytes reduced triacylglycerol but not diacylglycerol content, thus increasing the diacylglycerol/triacylglycerol ratio. In cardiomyocytes exposed to FA alone, GPAT inhibition reduced diacylglycerol but not triacylglycerol, thus decreasing the diacylglycerol/triacylglycerol ratio, prevented PKCδ activation and improved metabolic stress-stimulated glucose uptake. Changes in AMP-activated Protein Kinase activity failed to explain variations in metabolic stress-stimulated glucose uptake. Thus, LD formation regulates metabolic stress-stimulated glucose uptake in a manner best reflected by the diacylglycerol/triacylglycerol ratio.

## Introduction

Type 2 Diabetes Mellitus is a serious metabolic syndrome, the incidence of which is dramatically increasing worldwide. It makes up about 90–95% of total diabetes cases and is caused by insulin resistance combined with compromised activity of the pancreatic β-cells, which leads to hyperglycemia and subsequent glucose toxicity. Diabetic patients have increased myocardial infarction incidence and severity^1,2^. Stimulation of myocardial glucose uptake during ischemia improves the post-ischemic recovery of function and survival of the myocardium^3,4^. Thus, impairment of myocardial metabolic stress-stimulated glucose uptake could jeopardize the myocardial response to ischemic event and reperfusion injury.

A hallmark of type 2 Diabetes Mellitus is hyperlipidemia. Chronic exposure of cardiomyocytes to fatty acids (FA)^5^ or to triglyceride-rich lipoproteins particles^6^ results in marked inhibition of basal, insulin-stimulated and metabolic stress-stimulated glucose uptake. The mechanisms involved in the reduction of metabolic stress-stimulated glucose uptake by FA exposure involve inactivation of focal adhesion kinase (FAK)^7^ and chronic activation of protein kinase C δ (PKCδ)^8^. Chronic treatment with tetradecanoyl phorbol acetate (TPA) restores FAK activity and completely restores metabolic stress-stimulated glucose uptake in cardiac myocytes exposed to FA^7^. TPA is a diacylglycerol analog able to activate classic and novel PKC; however chronic exposure to TPA also triggers a marked downregulation of several PKC isoforms (PKCα, PKCδ and PKCε), and impairs the translocation of PKCδ to the membrane fraction^8^. TPA treatment also increases the biogenesis of lipid droplets. In the same way, chronic treatment with 5-aminoimidazole-4-carboxamide 1-β-D-ribofuranoside (AICAR), an AMP analog precursor capable of activating AMP-activated Protein Kinase (AMPK), results in an improvement of metabolic stress-stimulated glucose metabolism associated with a redirection of FA away from fatty acid oxidation and towards incorporation into lipid droplets^9^.

Lipid droplets are intracellular organelles that store neutral lipids within cells. They are mainly composed of a neutral lipid core containing mostly triacylglycerol and cholesteryl esters, surrounded by a phospholipid monolayer and lipid droplet-coating membrane proteins^10^. Skeletal muscle exhibits the “athlete’s paradox” as increased intramyocellular lipid concentration results in increased insulin responsiveness in trained skeletal muscle^11^. This phenomenon can be explained by the incorporation of fatty acid moieties in triacylglycerol within lipid droplets, instead of being turned into potentially toxic lipid derivatives. Triacylglycerol biogenesis is regulated by glycerol-3-phosphate acyl transferases (GPAT), which catalyze the acylation of glycerol-3-phosphate (G3P) into lysophosphatidic acid (LPA), and by diacylglycerol acyltransferases (DGAT), which converts diacylglycerol into triacylglycerol^12^. To understand the mechanisms for the protective effect of lipid droplets on glucose uptake, in this study we chronically inhibited lipid droplet biogenesis upstream (GPAT inhibition with FSG67^13^) and downstream (DGAT inhibition with either A922500^14^ or T863^15^) of the diacylglycerol synthesis step in cardiomyocytes exposed to either FA only or to FA with lipid droplets induction (TPA or AICAR). In this way, we expected to either decrease (GPAT inhibition) or increase (DGAT inhibition) the intracellular diacylglycerol concentration. We thus investigated how the effects of GPAT or DGAT inhibition on intracellular lipid management in cardiomyocytes influence glucose uptake, intracellular diacylglycerol concentration and PKCδ activation.

## Results

### Chronic inhibition of triacylglycerol synthesis reduced lipid droplets biogenesis

**Figure 1:**
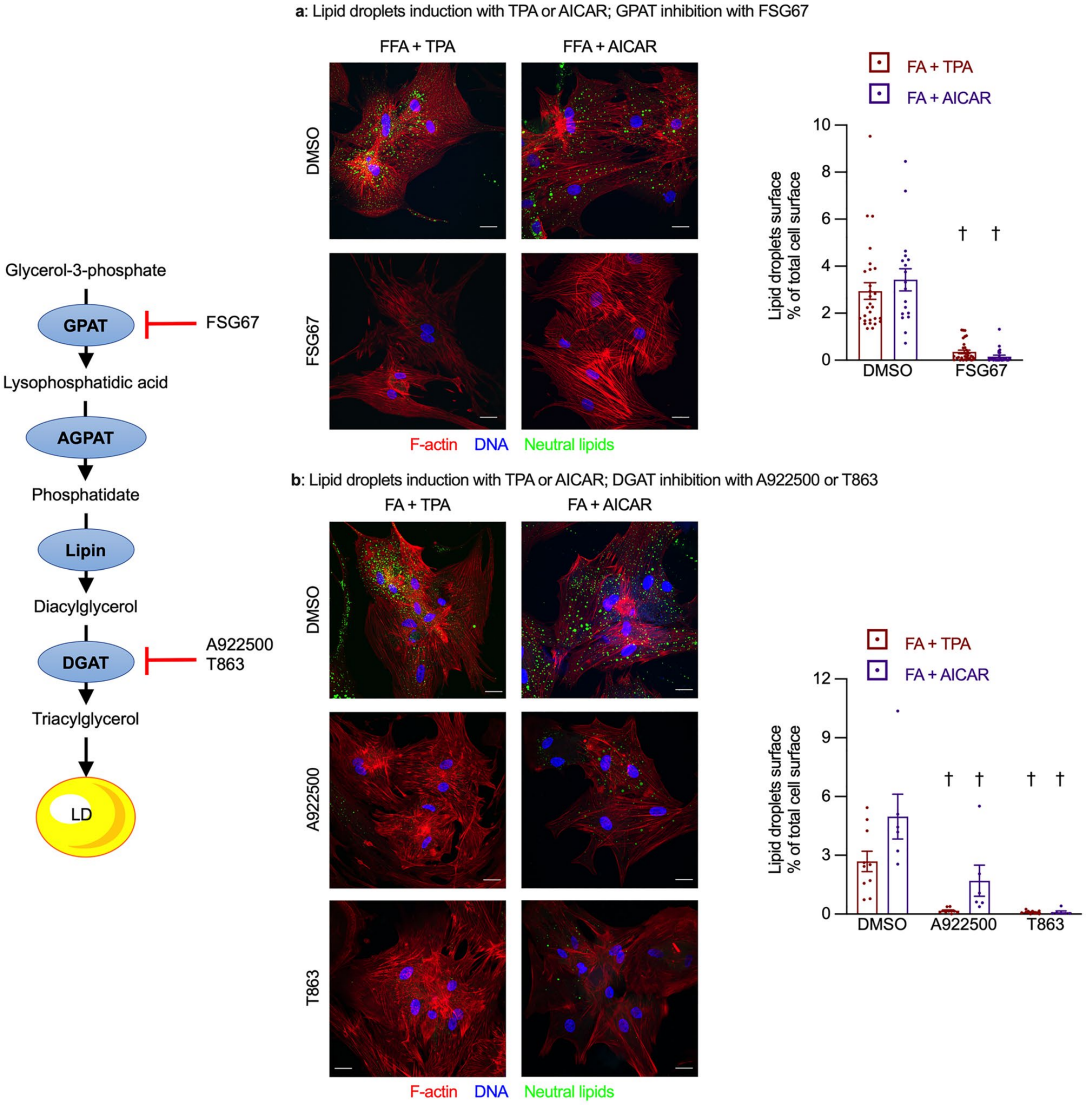
DGAT or GPAT inhibition reduce lipid droplet biogenesis. Primary cardiomyocytes were cultured for 7 days with 0.4 mM fatty acids (FA), 100 nM TPA or 0.2 mM AICAR and the GPAT inhibitor FSG67 (30 µM; panel **a**) or the DGAT inhibitors A922500 (1 µM) or T863 (100 nM) panel (**b**) or dimethyl sulfoxide as the vehicle control. Cells were fixed, permeabilized and stained for F-actin (red), DNA (blue) and neutral lipids (green) and the relative density of lipid droplets was measured (right panels). Scale bar 20 µm. Left panel shows the position of the inhibited enzymes in the triacylglycerol biosynthesis pathway. Results are shown as mean ± SEM; n = 6–32. †: significant effect (q < 0.05) of either the GPAT or the DGAT inhibitors.

We started by testing the effects of different perturbations in triacylglycerol metabolism on the biogenesis of lipid droplets. To do this, we inhibited enzymes acting either upstream (GPAT) or downstream (DGAT) of the diacylglycerol step in triacylglycerol biosynthesis and measured the effect on lipid droplet biogenesis (Fig. 1). We estimated the lipid droplet area as a percentage of total cell surface area. As previously observed^8,9^, chronic treatment with either TPA or AICAR induced lipid droplet biogenesis in cardiomyocytes exposed to FA, whereas very few lipid droplets were observed in cardiomyocytes exposed to FA alone. Chronic inhibition of either GPAT with FSG67 (Fig. 1a) or DGAT with A922500 or T863 (Fig. 1b) significantly reduced lipid droplet biogenesis in cardiomyocytes exposed to FA and lipid droplets inducers (TPA or AICAR), thus showing that triacylglycerol synthesis was required for lipid droplets induction and confirming the efficacy of the selected GPAT and DGAT inhibitors.

### GPAT inhibition improved glucose uptake in cardiomyocytes exposed to FA

We next sought to evaluate whether and how inhibition of lipid droplet biogenesis could influence glucose uptake. We first tested whether the two triacylglycerol synthesis inhibitors could influence glucose uptake in cardiomyocytes in the absence of FA or lipid droplets. Neither the GPAT inhibitor (FSG67), nor the DGAT inhibitors (A922500 and T863) modified basal, insulin- or metabolic stress-stimulated glucose uptake in cardiomyocytes not exposed to FA (Fig. S1).

**Figure 2:**
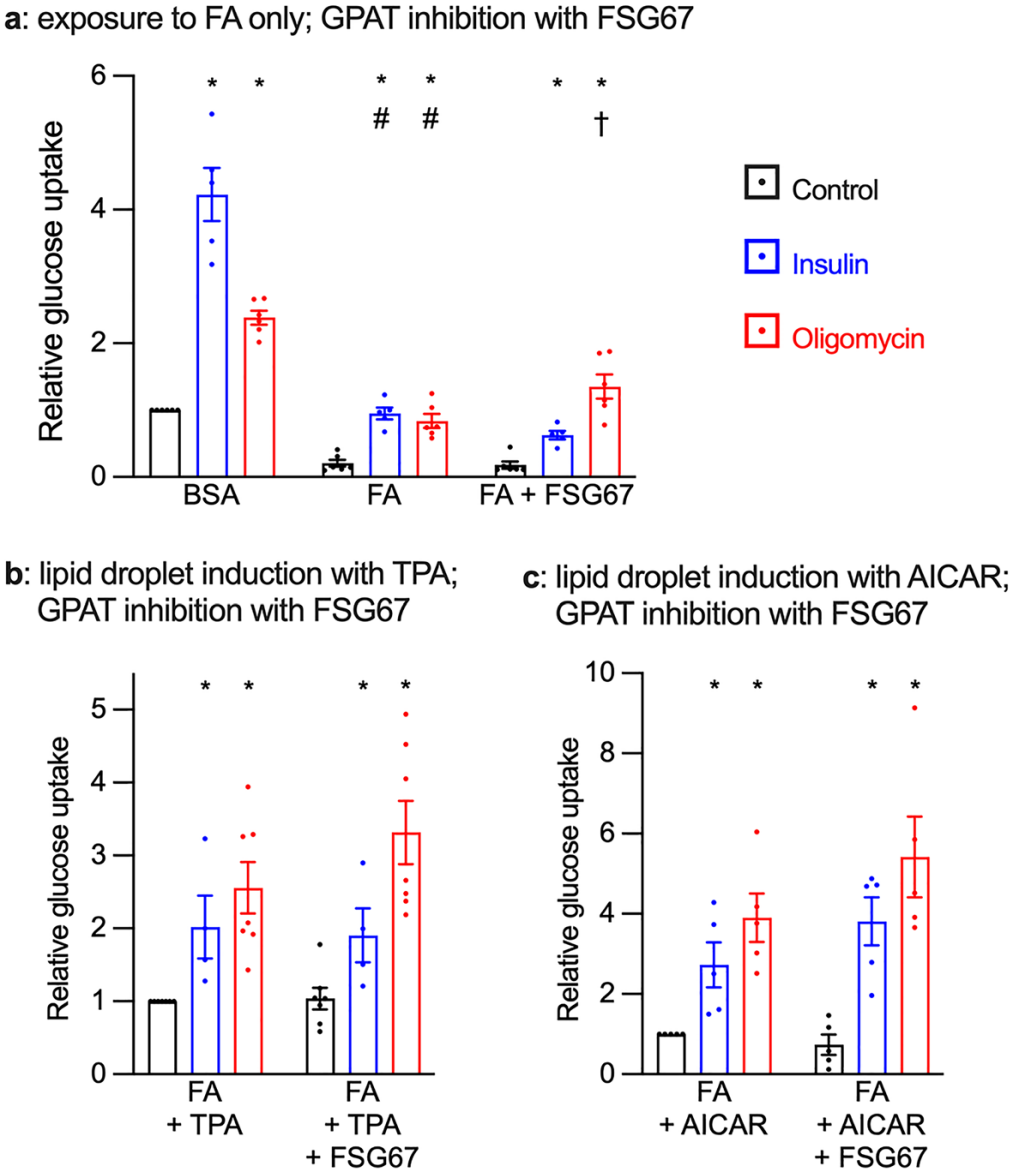
GPAT inhibition improves glucose uptake in cardiomyocytes exposed to FA: Primary cardiomyocytes were cultured for 7 days with or without 0.4 mM fatty acids (FA); lipid droplets were induced with either 100 nM TPA or 0.2 mM AICAR. Additional treatments were the GPAT inhibitor FSG67 (30 µM) or dimethyl sulfoxide as the vehicle control. Glucose uptake was then measured during 1 h exposure to either 1 µM insulin (blue bars), 1 µM oligomycin (red bars) or control (black bars). (**a**): effect of GPAT inhibition on glucose uptake in cardiomyocytes exposed to FA only. (**b**): effect of GPAT inhibition on glucose uptake in cardiomyocytes exposed to FA + TPA. (**c**): effect of GPAT inhibition on glucose uptake in cardiomyocytes exposed to FA + AICAR. Results are shown as mean ± SEM; n = 4–7. *: Significant effect (q < 0.05) of insulin or oligomycin; #: significant effect of FA. †: significant effect of the GPAT inhibitor FSG67.

Having thus established that inhibitors of triacylglycerol biosynthesis had no impact on glucose uptake in the absence of FA, we next investigated the effect of GPAT inhibition on glucose uptake in cardiomyocytes exposed to FA, with or without induction of lipid droplets. Chronic exposure to FA alone markedly impaired basal and stimulated glucose uptake (Fig. 2a), which is consistent with our previous observations^5,7–9^. GPAT inhibition in cardiomyocytes exposed to FA alone improved metabolic stress-stimulated glucose uptake (Fig. 2a). In cardiomyocytes with induced lipid droplets GPAT inhibition did not affect the glucose uptake in response to either insulin or oligomycin (Fig. 2b,c), although a trend towards an increase in metabolic stress-stimulated glucose uptake was observed. Basal and insulin-stimulated glucose uptake were not affected by GPAT inhibition. Overall, this demonstrates that inhibition of triacylglycerol synthesis upstream of the diacylglycerol step improved metabolic stress-stimulated glucose uptake in cardiomyocytes.

### GPAT inhibition improved AMPK signaling

**Figure 3:**
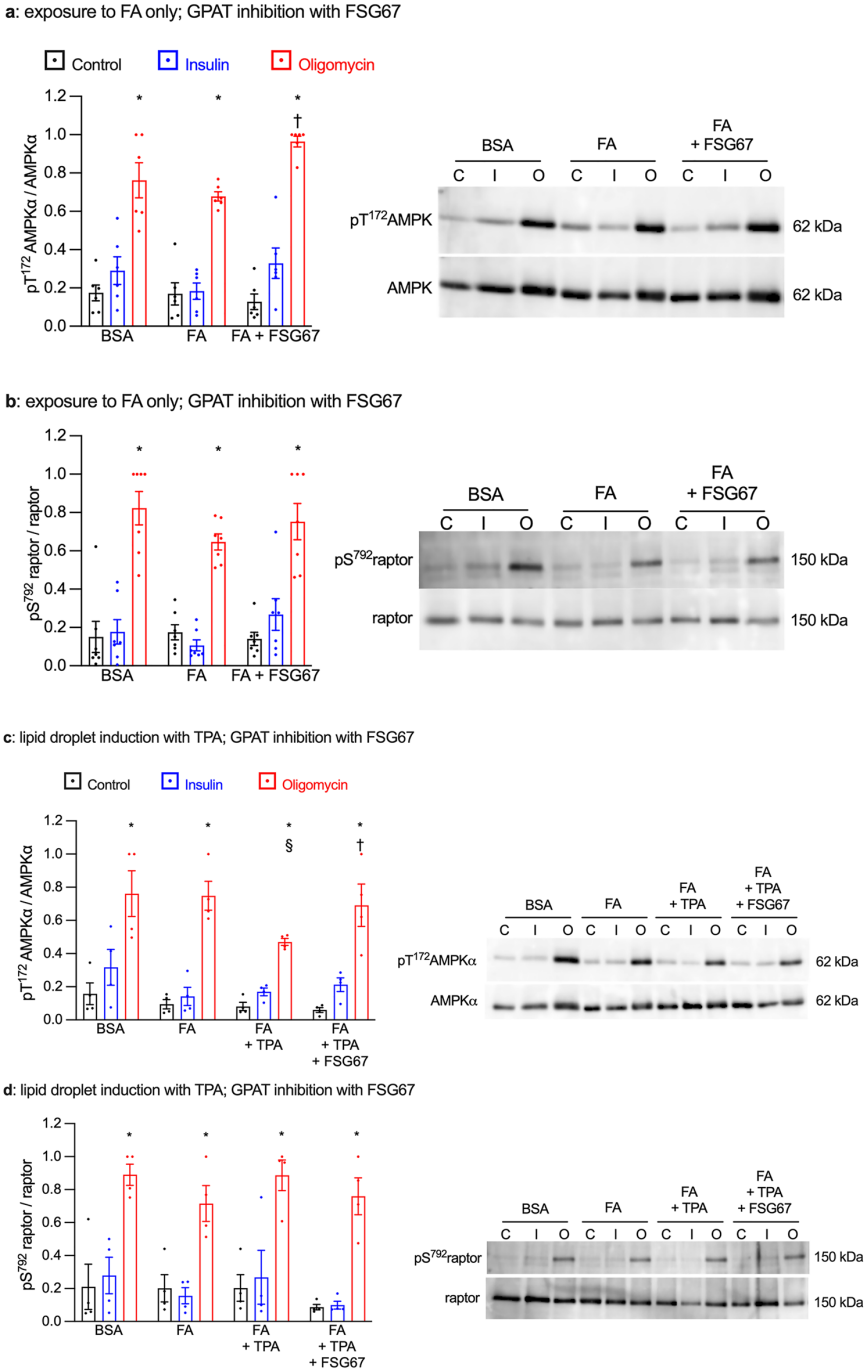

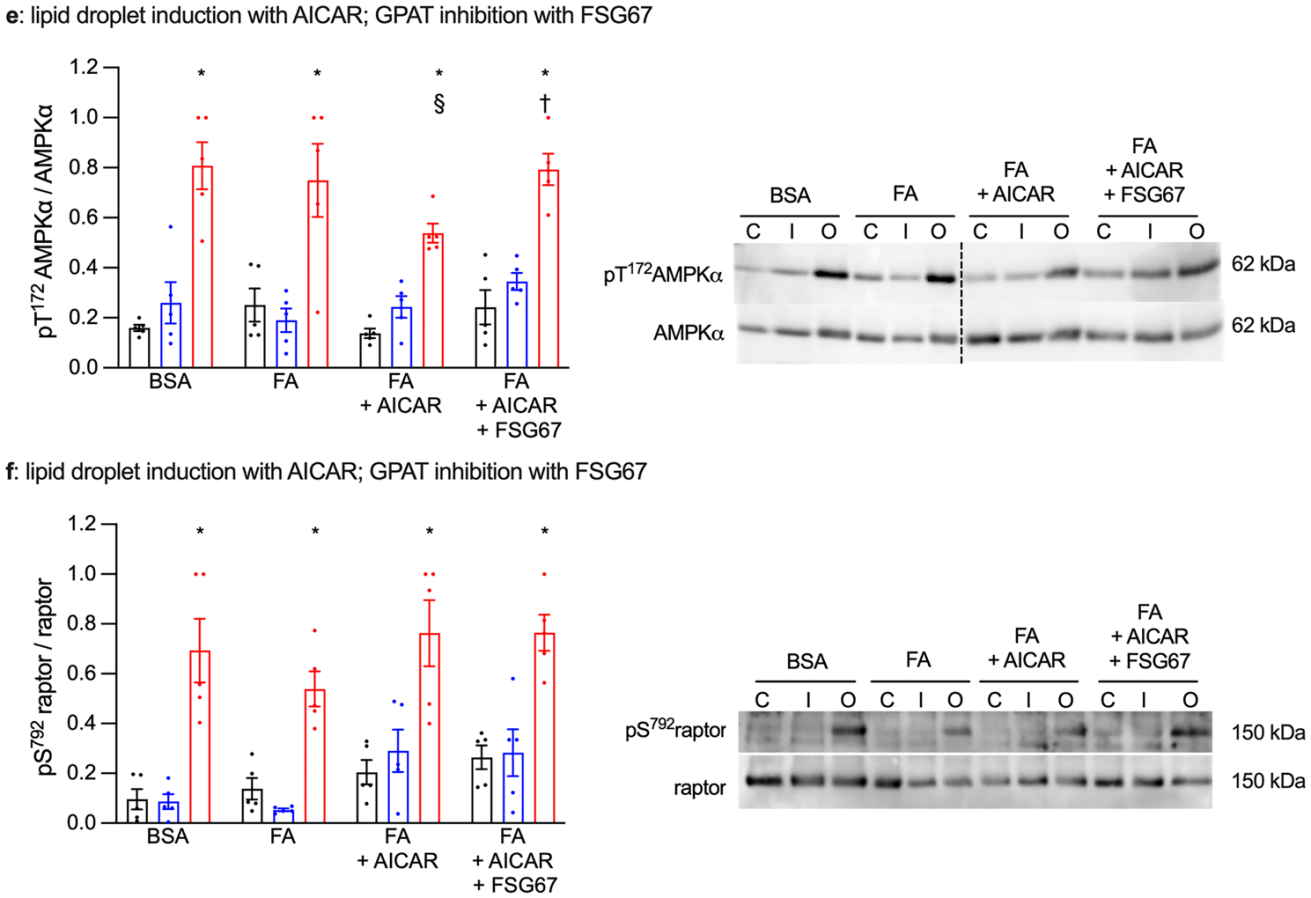
The presence of lipid droplets induced by TPA or AICAR reduces AMPK phosphorylation but not its activity. Primary cardiomyocytes were cultured for 7 days with or without 0.4 mM fatty acids (FA); lipid droplets were induced with either 100 nM TPA or 0.2 mM AICAR. Additional treatments were the GPAT inhibitor FSG67 (30 µM) or dimethyl sulfoxide as the vehicle control. Cardiomyocytes were then stimulated for 10 min with 1 µM insulin, for 20 min with 1 µM oligomycin or left unstimulated. Cells were extracted and submitted to western blot analysis to measure the expressions of phosphorylated (T^172^) and total AMPKα phosphorylation (panels **a**,**c**,**e**) and of phosphorylated (S^792^) and total raptor (panels **b**,**d**,**f**). The ratios of phosphorylated/total protein were than calculated and are displayed in the graphs. Right panels show western blots representative of the quantitative analysis in the graphs; the dashed line indicates where an image has been cut and spliced. Results are shown as mean ± SEM; n = 4–7. *: significant effect (q < 0.05) of oligomycin; §: significant effect of TPA or AICAR. †: significant effect of the GPAT inhibitor FSG67.

Since GPAT inhibition influenced only metabolic stress-stimulated glucose uptake, we focused our investigation on AMP-dependent Protein Kinase (AMPK) signaling. Indeed, AMPK is the energy gauge responsible for triggering the stress signal pathway leading to GLUT4 translocation^3,16^. Therefore, AMPK is activated in response to the intracellular ATP deprivation that occurs during ischemia in vivo or during oligomycin exposure in our in vitro model. We assessed AMPK activation by evaluating the phosphorylation of AMPKα subunit on the T^172^ residue and the phosphorylation of the AMPK substrate raptor on the S^792^ residue using phosphorylation-specific antibodies. Oligomycin-induced metabolic stress significantly increased both T^172^AMPKα and S^792^raptor phosphorylation in all experimental conditions (Fig. 3). GPAT inhibition in cardiomyocytes exposed to FA only increased T^172^AMPKα phosphorylation (Fig. 3a). Chronic treatment with either TPA or AICAR, which restored glucose transport and induced lipid droplet formation, significantly reduced T^172^AMPKα phosphorylation (Fig. 3c,e). In contrast, inhibition of lipid droplet biogenesis by GPAT inhibition restored T^172^AMPKα phosphorylation. These effects on Thr^172^AMPKα phosphorylation were not entirely reflected in its activity**,** as neither lipid droplet induction nor GPAT inhibition had any effect on S^792^raptor phosphorylation cardiomyocytes (Fig. 3b,d,f). The Rab GTPase-activating protein AS160 participates in the regulation of GLUT4 translocation and is also a substrate for AMPK, which phosphorylates AS160 on several residues recognized by the S/T-phosphorylated Akt substrate antibody^17,18^. Phosphorylation of AS160 was very variable, but generally followed a pattern similar to that of T^172^AMPKα (Fig S2a). Therefore, these data indicated that changes in AMPK phosphorylation and activity did not readily explain the improved metabolic stress-stimulated glucose uptake upon GPAT inhibition.

### DGAT inhibition reduces glucose uptake in cardiomyocytes with lipid droplets

**Figure 4:**
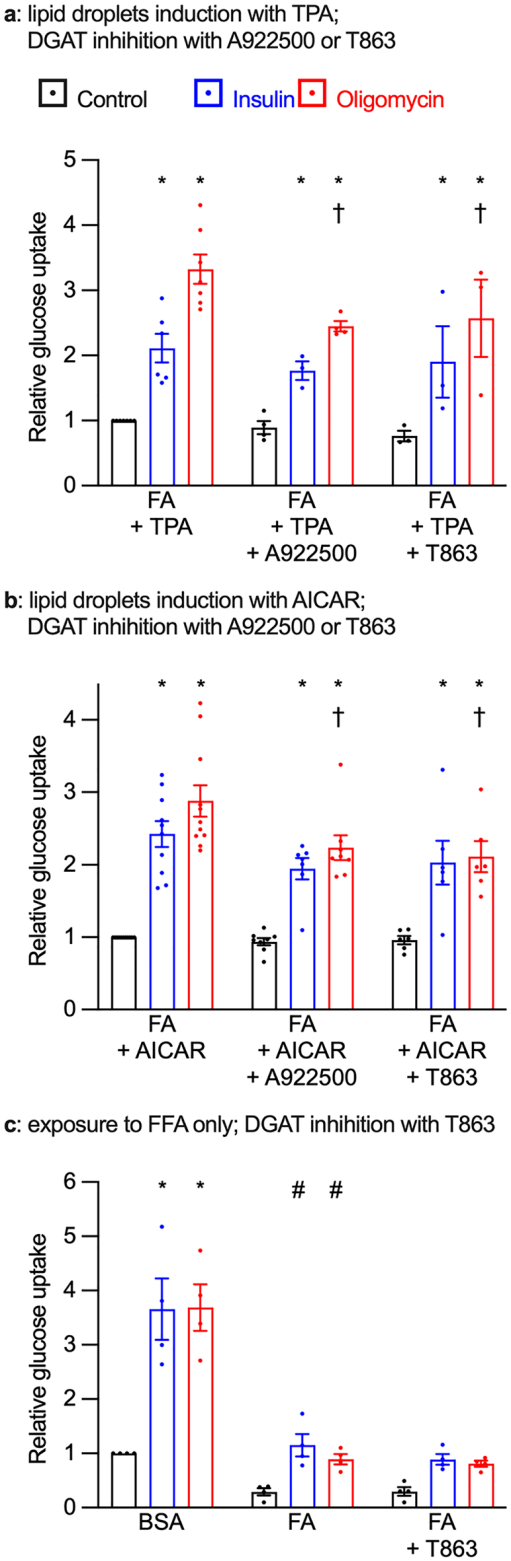
DGAT inhibition reduces glucose uptake in cardiomyocytes displaying lipid droplets. Primary cardiomyocytes were cultured for 7 days with 0.4 mM fatty acids (FA) in the presence or absence of 100 nM TPA or 0.2 mM AICAR. Additional treatments were the DGAT inhibitors A922500 (1 µM) or T863 (100 nM) or dimethyl sulfoxide as the vehicle control. Glucose uptake was then measured during 1 h exposure to either 1 µM insulin (blue bars), 1 µM oligomycin (red bars) or control (black bars). (**a**) Effect of DGAT inhibition on glucose uptake in cardiomyocytes exposed to FA + TPA. (**b**) Effect of DGAT inhibition on glucose uptake in cardiomyocytes exposed to FA + AICAR. (**c**) Effect of DGAT inhibition on glucose uptake in cardiomyocytes exposed to FA alone. Results are shown as mean ± SEM; n = 3–11. *: significant effect (q < 0.05) of insulin or oligomycin; #: significant effect of FA. †: significant effect of either DGAT inhibitor.

In a second approach we blocked lipid droplet biogenesis by inhibiting DGAT, the most downstream enzyme in the biosynthesis of triacylglycerol, with two structurally unrelated pharmacological inhibitors A922500 and T863. In contrast to GPAT inhibition, chronic DGAT inhibition significantly impaired the metabolic stress-stimulated, but not the insulin-stimulated, glucose uptake in cardiomyocytes exposed to either FA + TPA (Fig. 4a) or FA + AICAR (Fig. 4b). In cardiomyocytes exposed to FA only, chronic DGAT inhibition did not show any significant effect glucose uptake (Fig. 4c). Thus, in cardiomyocytes with induced lipid droplets biogenesis, DGAT inhibition has an effect on metabolic stress-stimulated glucose uptake opposite that of GPAT inhibition.

### DGAT inhibition improves AMPK phosphorylation but not its activity

**Figure 5:**
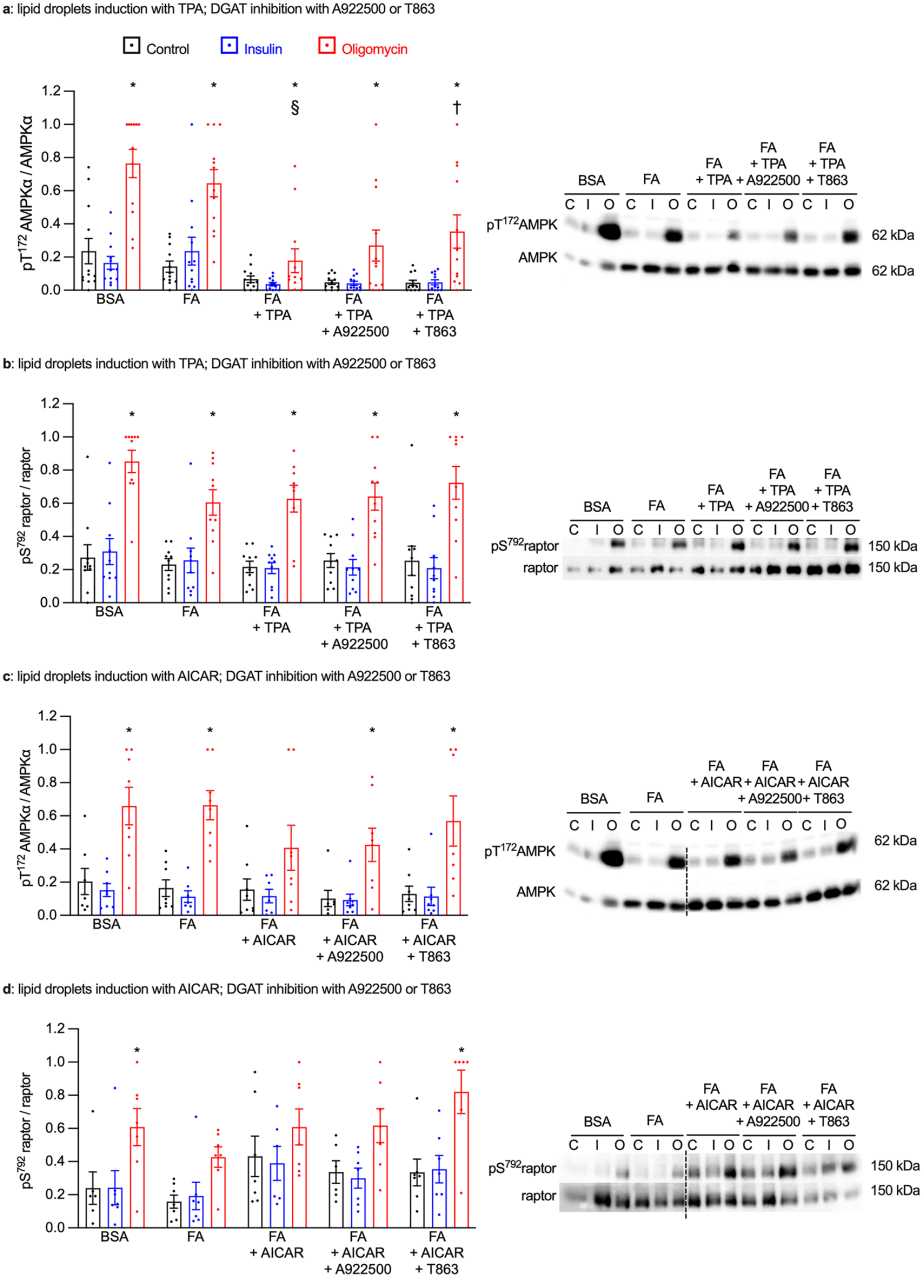
DGAT inhibition improves AMPK phosphorylation but not its activity. Primary cardiomyocytes were cultured for 7 days with or without 0.4 mM fatty acids (FA), 100 nM TPA (panels **a**,**b**) or 0.2 mM AICAR (panels **c**,**d**). Additional treatments were the DGAT inhibitors A922500 (1 µM) or T863 (100 nM) or dimethyl sulfoxide as the vehicle control. Cardiomyocytes were then stimulated for 10 min with 1 µM insulin, for 20 min with 1 µM oligomycin or left unstimulated. Cells were extracted and submitted to western blot analysis to measure the expressions of phosphorylated (T^172^) and total AMPKα phosphorylation (panels **a**,**c**) and of phosphorylated (S^792^) and total raptor (panels **b**,**d**). The ratios of phosphorylated/total protein were than calculated and are displayed in the graphs. Right panels show western blots representative of the quantitative analysis in the graphs; the dashed line indicates where an image has been cut and spliced. Results are shown as mean ± SEM; n = 6–12. *: significant effect (q < 0.05) of oligomycin; §: significant effect of TPA. †: significant effect of the DGAT inhibitor T863.

As again only metabolic stress-stimulated glucose uptake was affected by chronic DGAT inhibition, we assessed AMPK signaling. Although GPAT inhibition and DGAT inhibition had similar effects on lipid droplets biogenesis, they showed opposite effects on metabolic stress-stimulated glucose uptake in cardiomyocytes with induced lipid droplets. Nevertheless, similar to the results obtained with chronic GPAT inhibition, we observed a reduction of T^172^AMPKα phosphorylation in response to oligomycin in lipid droplet-displaying cardiomyocytes, which was partially restored by chronic DGAT inhibition (Fig. 5a,c). Again, none of the effects on Thr^172^AMPKα phosphorylation were reflected in its activity, as neither lipid droplet induction nor DGAT inhibition had any effect on S^791^raptor phosphorylation in FA + TPA or FA + AICAR-treated cardiomyocytes (Fig. 5b,d). Phosphorylation of AS160 was very variable, but generally followed a pattern similar to that of T^172^AMPKα (Fig. S2b). Thus, as with GPAT inhibition, changes in AMPK phosphorylation and activity upon DGAT inhibition did not correlate with metabolic stress-stimulated glucose uptake.

### GPAT inhibition prevented FA-induced PKCδ translocation to the membrane fraction

**Figure 6:**
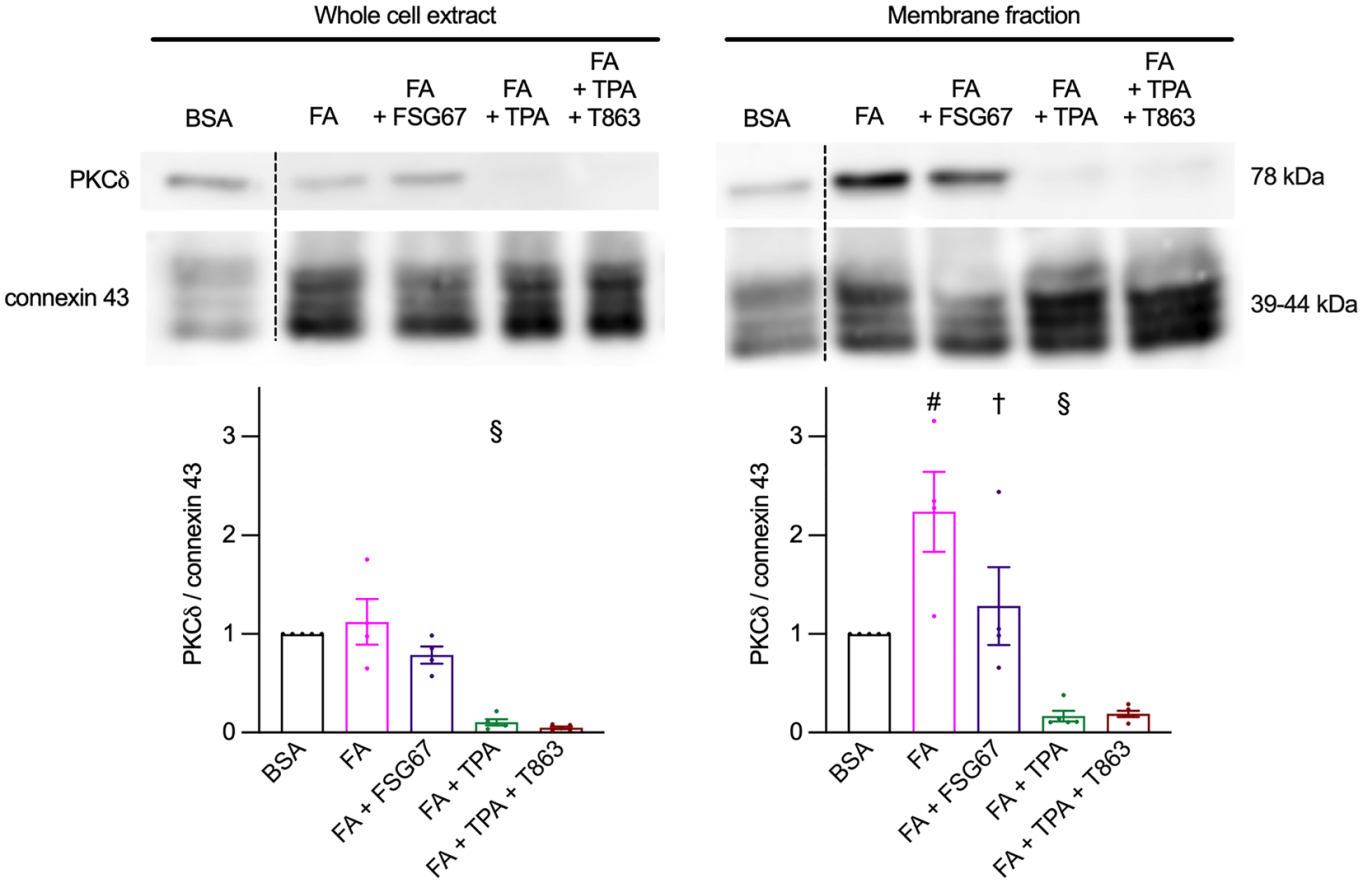
GPAT inhibition prevents PKCδ activation by FA. Primary cardiomyocytes were cultured for 7 days with or without 0.4 mM fatty acids (FA), 100 nM TPA and the GPAT inhibitor FSG67, the DGAT inhibitor T863 or dimethyl sulfoxide. The membrane fraction was then isolated, and whole cell extracts and membrane fractions submitted to western blot analysis of PKCδ and connexin 43 expression. Top panels show western blots representative of the quantitative analysis in the graphs below; the dashed line indicates where an image has been cut and spliced; for connexin 43 expression, membranes had been cut horizontally below the 55 kDa molecular weight marker before incubation with the primary antibody. Left: whole cell extracts; right: membrane fractions. Results are shown as mean ± SEM; n = 4–5. #: significant effect of FA. §: significant effect of TPA; †: significant effect of the GPAT inhibitor.

Novel PKCs are kinases which are completely dependent on diacylglycerol for their activity; once activated they translocate to the membrane fraction where they can phosphorylate several proteins^19^. In our previous study we identified the novel PKCδ as the isoform activated upon chronic FA exposure and, at least in part, involved in the reduction of glucose uptake^8^. We analyzed association of PKCδ with the membrane fraction of cardiomyocytes, characterized by the enrichment of the membrane-associated protein connexin 43^8^. Connexin 43 was selected as a convenient marker of the membrane fraction, being present in cardiomyocytes in both the plasma membrane and mitochondria^20^, two compartments to which PKCδ translocates upon activation^21^. We focused our analysis on a subset of conditions showing significant differences in metabolic stress-stimulated glucose uptake in response to inhibition of lipid droplet biogenesis, i.e. GPAT inhibition in cardiomyocytes exposed to FA, and DGAT inhibition (T863) in cardiomyocytes exposed to FA + TPA (Fig. 6). PKCδ expression (left panel) remained unchanged upon chronic FA exposure with or without GPAT inhibition. In contrast, as previously observed, chronic TPA treatment almost abolished PKCδ expression (Fig. 6). Chronic FA exposure induced translocation of PKCδ to the membrane fraction (right panel). However, GPAT inhibition significantly blunted the FA-induced translocation of PKCδ. Chronic TPA treatment abolished PKCδ translocation in cardiomyocytes exposed to FA; DGAT inhibition in addition to chronic TPA treatment had no effect. Thus, the improvement of metabolic stress-stimulated glucose uptake by GPAT inhibition in FA-exposed cardiomyocytes (Fig. 2a) was associated with prevention of PKCδ translocation.

### GPAT and DGAT inhibition both reduced intracellular diacylglycerol

**Figure 7:**
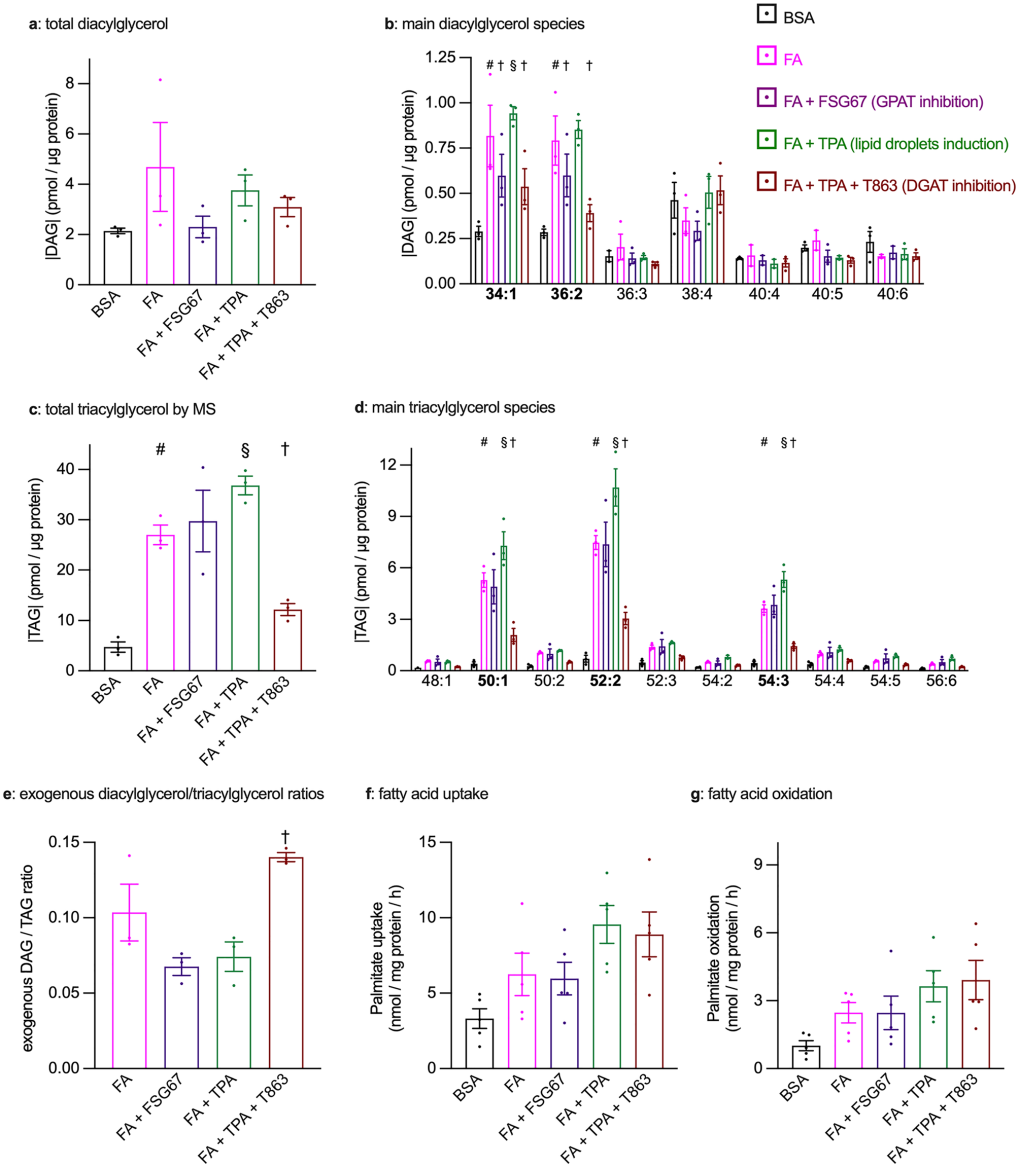
Analysis of cellular diacylglycerol and triacylglycerol levels and of FA metabolism in cardiomyocytes under GPAT or DGAT inhibition. Primary cardiomyocytes were cultured for 7 days with or without 0.4 mM fatty acids (FA), 100 nM TPA and the GPAT inhibitor FSG67, the DGAT inhibitor T863 or dimethyl sulfoxide. Panels (**a–e)** Total cellular lipids were then extracted and analyzed by mass spectrometry. (**a**) Total diacylglycerol (DAG). (**b**) Diacylglycerol species identified in all samples by MS. Species are identified by total acyl chain length and total number of double bonds; bold characters identify palmitate and/or oleate-based species. (**c**) Total triacylglycerol (TAG). (**d**) Detail of the 10 most prominent triacylglycerol species. Species are identified by total acyl chain length and total number of double bonds; bold characters identify palmitate and/or oleate-based species. (**e**): Exogenous diacylglycerol/triacylglycerol ratios (DAG/TAG). Panels (**f**–**g**) After the 7 days culture period as above, palmitate uptake and oxidation were measured. Results are shown as mean ± SEM; n = 3–5. #: significant effect of FA. §: significant effect of TPA; †: significant effect of the GPAT or the DGAT inhibitor.

To determine how changes in intracellular diacylglycerol and triacylglycerol contents could explain changes in glucose uptake and PKCδ translocation, we performed high-performance thin-layer chromatography (HPTLC) and shotgun mass spectrometry (MS) analyses of total cellular lipids with the same set of conditions as the PKCδ translocation experiments. Diacylglycerols were barely detectable, and not quantifiable by HPTLC. In MS analyses, exposure to FA led to an increased intracellular content of total diacylglycerol as compared to cardiomyocytes exposed to BSA alone, while GPAT inhibition reduced diacylglycerol content during FA exposure (Fig. 7a,b) Surprisingly, TPA treatment failed to reduce diacylglycerol content despite induced lipid droplet biogenesis. Furthermore, DGAT inhibition did not increase intracellular amount of diacylglycerol in FA + TPA-treated cardiomyocytes. Looking into the different diacylglycerol (DAG) species, we discovered that DAG(34:1) (comprising one palmitoyl and one oleyl chain) and DAG(36:2) (comprising two oleyl chains) were the most predominant in cardiomyocytes chronically exposed to FA, which were oleate and palmitate. In contrast, the most prominent diacylglycerol in control cardiomyocytes was DAG(38:4) (Fig. 7b). Both DAG(34:1) and DAG(36:2) were significantly increased in FA-exposed cardiomyocytes, and both were significantly reduced when GPAT activity was chronically inhibited in FA + FSG67-treated cardiomyocytes (Fig. 7b). Again, chronic TPA treatment failed to reduce different diacylglycerol species concentrations. Remarkably, when DGAT activity was inhibited in FA + TPA + T863-treated cardiomyocytes, thus preventing lipid droplets biosynthesis, the palmitate- and oleate-based diacylglycerols were significantly reduced (Fig. 7b), although there was no difference in total diacylglycerol amounts (Fig. 7a). In this condition we observed a significant increase in palmitate- and oleate-based phosphatidyl choline (Fig. S3), reflected by a similar trend in total palmitate- and oleate-based phospholipids. Thus, prevention of diacylglycerol accumulation by GPAT inhibition in FA-exposed cardiomyocytes correlated with reduced PKCδ translocation (Fig. 6) and improved metabolic stress-stimulated glucose uptake (Fig. 2a).

### Only DGAT inhibition prevented triacylglycerol accumulation

MS and HPTLC analyses of cellular triacylglycerol yielded similar results (Fig. 7c and S4). Triacylglycerol increased upon chronic FA exposure but remained unexpectedly high following GPAT inhibition. TPA co-treatment with FA pushed the triacylglycerol levels significantly higher, whereas DGAT inhibition in this condition markedly reduced triacylglycerol levels. Moreover, we discovered that among the most abundant triacylglycerol (TAG) isoforms, TAG 50:1 (2 palmitoyl + 1 oleyl chains), TAG 52:2 (1 palmitoyl + 2 oleyl chains) and TAG 54:3 (3 oleyl chains) are the ones whose concentration was significantly influenced by lipid droplet formation (Fig. 7d).

Because neither diacylglycerol nor triacylglycerol contents readily explained the observed changes in glucose uptake, we surmised that the differential management of exogenous FA, expressed as the ratio of diacylglycerol to triacylglycerol, might rather be a determinant of glucose uptake. The ratio of exogenous diacylglycerol/triacylglycerol was calculated for cardiac myocytes exposed to FA; we observed that the conditions that reduced the diacylglycerol/triacylglycerol ratio upon FA exposure (GPAT inhibition or TPA chronic treatment) improved the metabolic stress-stimulated glucose uptake. Conversely, the increased diacylglycerol/triacylglycerol ratio resulting from DGAT inhibition is associated with impaired glucose uptake during metabolic stress in cardiomyocytes exposed to FA + TPA (Fig. 7e). Therefore, metabolic stress-stimulated glucose uptake was reduced when the diacylglycerol/triacylglycerol ratio was increased and conversely.

### DGAT or GPAT inhibition did not affect FA uptake or oxidation

To gain further insight into possibly relevant changes in FA metabolism, we measured palmitate uptake and oxidation in conditions showing significant differences in metabolic stress-stimulated glucose uptake in response to inhibition of lipid droplet biogenesis, i.e. GPAT inhibition in cardiomyocytes exposed to FA, and DGAT inhibition (T863) in cardiomyocytes exposed to FA + TPA. As previously observed^8,9^, chronic FA exposure tended to increase both palmitate uptake (Fig. 7f) and oxidation (Fig. 7g), although the data failed to reach statistical significance. GPAT inhibition however did not modify either rate of FA metabolism. Chronic TPA treatment further increased palmitate uptake in cardiomyocytes exposed to FA, consistent with the marked accumulation of lipid droplets. However, DGAT inhibition, which markedly reduced lipid droplets, did reduce neither palmitate uptake nor its oxidation.

## Discussion

In previous studies we observed that the induction of lipid droplets was associated with the restoration of stimulated glucose uptake in cardiomyocytes exposed to FA^8,9^. We hypothesized that it was the accumulation of diacylglycerol in FA-exposed cardiomyocytes that triggered PKCδ activation and thereby inhibited glucose uptake^8^. In this context, accumulation of triacylglycerol-rich lipid droplet would lead to diacylglycerol consumption for triacylglycerol synthesis. This should prevent PKC activation and thus indirectly preserve glucose uptake.

In the present study, we investigated whether lipid droplets directly could contribute to protect glucose uptake in cardiomyocytes exposed to fatty acids. For this purpose, we prevented their constitution through inhibition of enzymes of the triacylglycerol biosynthesis pathway, GPAT and DGAT. By acting at two distinct levels of the triacylglycerol biosynthetic pathway, we expected to differentially modify diacylglycerol accumulation: GPAT inhibition should prevent, but DGAT inhibition worsens, diacylglycerol accumulation.

The main findings of this study can be summarized in 6 parts:

1. GPAT and DGAT inhibitions showed different effects on glucose uptake between cardiomyocytes exposed to FA only and cardiomyocytes displaying LD in presence of FA
2. GPAT inhibition in cardiomyocytes exposed to FA alone reduces cellular diacylglycerol, prevents PKCδ activation and improves metabolic stress-stimulated glucose uptake, thus partially supporting the hypothesis formulated above and in our previous paper^8^.
3. Lipid droplet biogenesis in cardiomyocytes does not reduce diacylglycerol levels, suggesting that additional mechanisms are at play. In cardiomyocytes with lipid droplets, GPAT inhibition has little effect on glucose uptake while DGAT inhibition impairs metabolic stress-stimulated glucose uptake, again suggesting the existence of additional mechanisms.
4. Metabolic stress-stimulated glucose uptake seems to be negatively impacted by an increased diacylglycerol/triacylglycerol ratio, and vice versa*.*
5. Manipulations of lipid droplet biogenesis have a specific impact on metabolic stress-stimulated glucose uptake, without affecting the response to insulin, showing that the responses to insulin and metabolic-stress are differentially affected by alterations of intracellular fatty acids management.
6. Changes in AMPK activity, if any, do not seem to explain the variations in metabolic stress-stimulated glucose uptake, as readouts of AMPK activity vary in opposite directions to metabolic stress-stimulated glucose uptake when lipid droplets biosynthesis is induced by TPA or AICAR or reduced by DGAT inhibition.

Chronic FA exposure presumably induces PKCδ activation by a mechanism involving the generation of diacylglycerol, known to be the physiological activator of classic and novel PKC. In several models of cultured cardiomyocytes, provision of FA results in increased cellular contents of diacylglycerol^22–24^ and activation of PKCδ^22^. We observed that chronic GPAT inhibition, by reducing intracellular diacylglycerol concentration, prevented PKCδ activation and improved metabolic stress-stimulated glucose uptake in cardiomyocytes exposed to FA alone. In our previous study^8^ we showed that chronic treatment with the PKCδ specific inhibitor rottlerin was able to improve insulin and metabolic stress-stimulated glucose uptake in cardiomyocytes exposed to FA. Inhibition of PKCδ translocation has been shown to reduce ischemia and reperfusion-induced myocardial dysfunction and resulted in an improved regeneration of intracellular ATP^25^, while the specific PKCδ inhibitor KAI-9803 ameliorated injury associated with ischemia and reperfusion in animal models of acute myocardial infarction^26^. However, PKCδ activation only partially explains the effect of FA, as we did not observe a complete restoration of glucose uptake despite normalization of PKCδ translocation. This suggests that PKCδ activation represents only a part of the complex mechanisms by which FA impair glucose uptake in cardiomyocytes.

The primary aim of this study was to evaluate if lipid droplet biogenesis protected glucose uptake against inhibition by FA by reducing the intracellular amount of bioactive lipid intermediates such as diacylglycerol, thereby preventing PKCδ activation. In our previous studies, we showed that chronic TPA treatment induced an abrogation of several PKC isoforms and a significant inhibition of PKCδ translocation to the membrane fraction^8^. However, chronic AICAR treatment, which also induced lipid droplet formation, failed to inhibit PKCδ translocation^9^. Lipidomics analysis indicated that lipid droplet formation was not accompanied by a significant reduction of intracellular diacylglycerol. This ruled out lipid droplet biogenesis itself as the driver of PKCδ activity prevention in cardiomyocytes exposed to FA + TPA. Instead, TPA treatment on these cells had the effect of abrogating PKCδ expression. Therefore, this indicates that lipid droplet formation occurring as a result of chronic AICAR and TPA treatments on cardiomyocytes has a protective role on glucose uptake not linked to PKCδ inhibition.

Lipidomics analysis showed that exogenous FA increased intracellular diacylglycerol content; specifically, the main diacylglycerol species found in FA-exposed cardiomyocytes were oleate- and palmitate-based. As expected, chronic GPAT inhibition significantly reduced oleate- and palmitate-derived diacylglycerols while other diacylglycerol species were reduced without reaching statistical significance, confirming that exogenous FA might be responsible for PKCδ activation. Surprisingly, lipid droplet biogenesis did not reduce diacylglycerol content in cardiomyocytes exposed to FA + TPA; possibly the increased FA uptake induced by TPA^8^ (Fig. 7f) is not entirely compensated by increased incorporation of fatty acyl moieties into triacylglycerol. Also, no increase in the amount of cellular diacylglycerol was observed when DGAT activity was chronically inhibited. Taken together, these results suggest that the protective effect of lipid droplets on glucose uptake was not directly related to diacylglycerol accumulation and consumption.

Similar observations had been made in vivo in mice, wherein myocardial diacylglycerol was increased following high fat diet but not further increased by DGAT1 deficiency^27^. Conversely, myocardial overexpression of DGAT1 was shown to reduce diacylglycerol concentration and protected the heart from lipotoxicity^28^.

DGAT1 overexpression in skeletal muscles protected mice from high fat diet-induced insulin resistance, while DGAT1 deficiency diminished insulin sensitivity^29^. However, in our model, chronic GPAT or DGAT inhibition in cardiomyocytes displaying lipid droplets did not influence insulin-stimulated glucose uptake and only DGAT inhibition impaired metabolic stress-stimulated glucose uptake. This discrepancy might pertain to different metabolic properties of skeletal muscle vs. myocardium. To the best of our knowledge, no study investigated the involvement of DGAT activity in insulin sensitivity of the myocardium.

As expected, cardiomyocytes displaying lipid droplets also showed increased intracellular triacylglycerol concentration. However, both mass spectrometry- and chromatography-based lipidomics showed significant levels of triacylglycerol were also present in FA-exposed cardiomyocytes even though very few lipid droplets were observed. In addition, with chronic GPAT inhibition in FA-exposed cardiomyocytes, triacylglycerol level remained elevated despite a decrease of diacylglycerol concentration. These observations suggest that significant triacylglycerol amounts may exist in cardiomyocytes outside of lipid droplets as identified by our staining method.

How could substantial amount of triacylglycerol accumulate in cardiomyocytes exposed to FA despite GPAT inhibition ? In the heart, triacylglycerol synthesis is mainly regulated by GPAT1 and GPAT3, localized in the mitochondria and in the endoplasmic reticulum, respectively^12^; both GPAT isoforms are inhibited by FSG67. Since the other triacylglycerol biosynthesis intermediate phosphatidic acid, which is also downstream of GPAT, did not show much variation, one hypothesis could be that an alternative pathway bypassing GPAT, such as acylation of dihydroxyacetone phosphate followed by the reduction to lysophosphatidic acid, can induce triacylglycerol formation even when GPAT activity is inhibited. Indeed, Kojta et al.^30^ showed how GPAT silencing in skeletal muscle markedly reduced diacylglycerol content but with a much lesser effect on triacylglycerol.

Significant changes in cellular diacylglycerol and triacylglycerol concentrations were only observed for the palmitate- and oleate-based species, which were dominant in FA-containing conditions, but minorized in the BSA control condition. This suggests that exogenous FA and their derivatives are the principal effectors of lipid-induced metabolic modulation in cardiomyocytes, in line with the very limited capacity of cardiomyocytes for the biosynthesis of fatty acids. Similar to the situation with the amounts of diacylglycerol, we could not discern a direct correlation, positive or negative, between cellular triacylglycerol contents and glucose uptake. However, there seems to be a good inverse correlation between metabolic stress-stimulated glucose uptake and the exogenous diacylglycerol/triacylglycerol ratio, suggesting that this ratio might be more important than the actual concentration of either of its determinants in regulating glucose uptake.

In a previous study we observed that either stimulation or complete abolition of FA oxidation could improve metabolic stress-stimulated glucose uptake^5^. In the present study however, neither DGAT nor GPAT inhibition had any effect on FA oxidation, arguing against modulation of FA oxidation playing a role in the effects of these maneuvers on glucose uptake.

Extracellular lipids may interact with glucose uptake regulation by inducing disassembly and inhibition of the vacuolar-type H^+^-ATPase (v-ATPase)^31^. This results in further stimulation of FA uptake and inhibition of insulin-stimulated GLUT4 translocation both in cultured cardiomyocytes and in ex vivo cardiomyocytes obtained from rats fed a high-fat diet^31,32^. Although LD biogenesis could prevent v-ATPase by sequestering intracellular FA, we do not think that this mechanism is involved in the protective effect of LD on metabolic stress-stimulated glucose uptake for two reasons. First, v-ATPase reactivation restored insulin-stimulated glucose uptake in palmitate-exposed cardiomyocytes, whereas manipulation of LD biosynthesis in our study only impacted metabolic stress-stimulated, not insulin-stimulated glucose uptake. Second, induction of LD biosynthesis resulted in a stimulation of FA uptake rather than an inhibition (Fig. 7f), as should have been the case with v-ATPase reactivation^33^.

Activation of the AMPK complex mediates the stimulation of myocardial glucose uptake by metabolic stress in vivo^3^, ex vivo^16^ and in vitro^9^. Thus, we investigated AMPK signaling, assessed at two levels: phosphorylation of AMPKα on T^172^ and phosphorylation of raptor (an AMPK substrate not involved in the regulation of glucose uptake) on S^792^. We have found an interesting relationship between lipid droplet biogenesis and AMPKα phosphorylation, since we observed that both chronic treatments inducing lipid droplet formation (TPA and AICAR) reduced T^172^ AMPKα phosphorylation, while chronic GPAT or DGAT inhibition restored it. Nevertheless, these effects were not reflected on S^792^ raptor phosphorylation, used as a readout for AMPK activity. Raptor phosphorylation was previously shown to better correlate with metabolic-stress stimulated glucose uptake than AMPKα phosphorylation^9^. This may suggest that the regulatory mechanisms of glucose uptake impacted by variations in lipid droplet metabolism are either downstream or independent of AMPK activation. However, as far as we know, raptor is not directly involved in the regulation of glucose uptake in cardiomyocytes^34^ and we cannot exclude that other pathways may be differentially activated by AMPK as compared to raptor phosphorylation.

In contrast to raptor, the phosphorylation of AS160 is known to be required for translocation of GLUT4 to occur in response to metabolic stress, and AS160 is a direct target of AMPK^17,18^. Indeed, the variations of AS160 phosphorylation with variations in lipid droplet metabolism generally matched the changes in AMPKα phosphorylation. However, these variations go in opposite directions as compared to glucose uptake, and therefore cannot explain the effects of lipid metabolism on metabolic stress-stimulated glucose uptake.

Being essential for the maintenance of cellular energy homeostasis, AMPK is also involved in the regulation of lipid metabolism in cardiomyocytes. AMPK can modulate lipid droplet recycling and dispersion via perilipin phosphorylation^35,36^. AMPK, by regulating hormone-sensitive lipase and perilipin activity within lipid droplets, induces triacylglycerol hydrolysis in adipocytes and L6 myoblasts^37,38^. However, we are not aware of any evidence regarding a reverse direct effect of lipid droplet metabolism on AMPK regulation. The only observation in the literature similar to ours was made in hepatocytes of rats on a high-fat diet, wherein increases in both GPAT expression and lipid droplet density were also associated with reduced AMPK phosphorylation^39^, suggesting that GPAT biosynthetic activity could directly influence AMPK activation.

Some limitations of this study deserve comments. Due to technical reasons, the primary limitation of this study was the low number of conditions and replicates used for mass spectrometry-based lipid analysis. We focused on the most significant conditions pertaining to changes in metabolic stress-stimulated glucose uptake. Nevertheless, a clear signal was obtained in these albeit limited conditions.

Another limitation was our inability to identify mechanisms downstream of AMPK involved in glucose metabolism that are affected by alterations of lipid droplet metabolism.

The last limitation was the use of the ATPase inhibitor oligomycin as a surrogate for ischemia in vitro. Ischemia in vivo impacts the mitochondrial function in a much more complex manner than simply shutting off ATPase activity. However simulated ischemia in vitro takes a much longer time to act than in vivo, long enough that changes in protein expression, such as overexpression of GLUT1, can occur and confound the interpretation of glucose uptake data^40^. Therefore, oligomycin remains a convenient way to achieve ischemia-like energy depletion and AMPK activation without changing protein expression.

The experimental model used herein also deserves a comment. Adult rat cardiomyocytes in long-term primary culture under high FCS conditions undergo dedifferentiation and redifferentiation, resulting after 7–8 days in a phenotype with high myofibrillar organization, contractility^41^ and a glucose transport response to insulin and metabolic stress almost identical to that observed in freshly isolated cardiomyocytes^42,43^ despite extensive cytoskeletal reorganization leading to the loss of the rod-like shape. In addition, inclusion of 9-cis retinoic acid to the culture medium markedly reduces the extent of transient dedifferentiation and of subsequent hypertrophy^42–44^. We therefore believe this experimental model to be valid for investigations on the impact of chronic metabolic interventions on glucose metabolism in cardiomyocytes.

**Figure 8:**
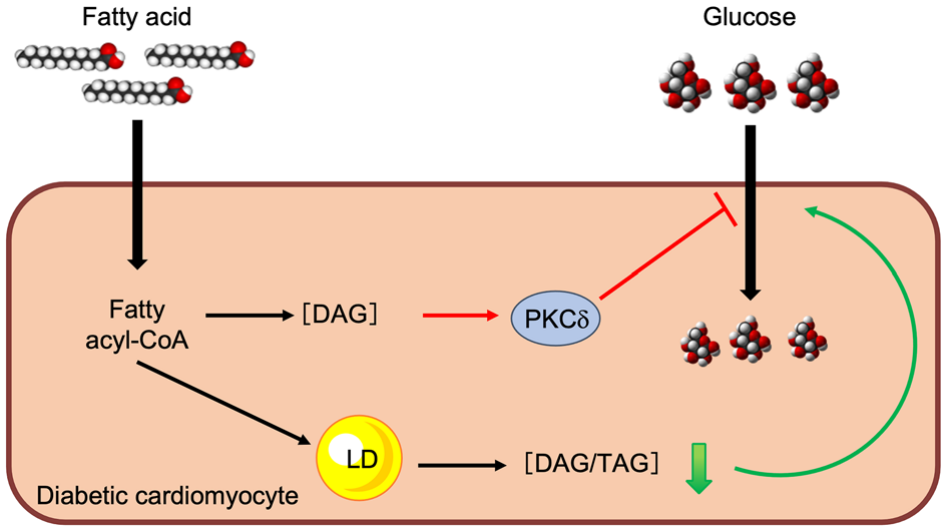
Conclusions. Chronic fatty acids (FA) exposure of cardiomyocytes increases intracellular diacylglycerol (DAG) concentration, thereby inducing PKCδ activation and impairing metabolic stress-stimulated glucose uptake. Lipid droplet formation reduces the diacylglycerol/triacylglycerol (DAG/TAG) ratio, thus preserving glucose uptake despite diacylglycerol accumulation, suggesting a protective effect by lipid droplets independent of PKCδ activity. Red arrows: mechanisms impairing metabolic stress-stimulated glucose uptake; green arrows: mechanisms preserving metabolic stress-stimulated glucose uptake.

In conclusion (Fig. 8), we showed that chronic FA exposure on cardiomyocytes increased intracellular diacylglycerol concentration, thereby inducing PKCδ activation and impairing metabolic stress-stimulated glucose uptake. Instead, boosting lipid droplet biogenesis protects glucose uptake in FA-exposed cardiomyocytes in a mechanism independent from diacylglycerol levels. Critically, we found that the intracellular diacylglycerol/triacylglycerol ratio of oleate- and palmitate-derived FA is inversely related to glucose uptake.

## Material and method

### Rat cardiomyocytes culture

Male Sprague Dawley rats (100–200 g) obtained from Janvier Labs (France) were deeply anesthetized by an intraperitoneal injection of ketamine (100 mg/kg) and xylazine (10 mg/kg) and the hearts were harvested under deep coma. All the methods were carried out in accordance with relevant institutional guidelines and regulations. The protocol was approved by the Canton of Geneva Committee for Animal Experimentation (authorizations # GE/210/17, GE/60/19 and GE153). All the experiments were carried out in accordance with ARRIVE guidelines.

Cardiomyocytes were isolated as previously described^45^ by retrograde perfusion of the hearts with collagenase (type II; Worthington; 120 IU/ml) and hyaluronidase (1% w/v)^42,46^. Cardiomyocytes were separated from non-myocyte cardiac cells by pre-plating the whole cell suspension for 90 min on untreated plastic, to which non-myocyte cells, but not cardiomyocytes, readily adhere. Cardiomyocytes were plated in M199 medium containing 5.5 mM glucose supplemented with 5 mM creatine, 2 mM L-carnitine, 5 mM taurine, 100 μM cytosine-β-D- arabinofuranoside, 100 nM 9-cis retinoic acid, 10 nM triiodothyronine and 20% fetal calf serum. Dishes were previously coated with 0.1% gelatin for 4 h and incubated overnight with complete culture medium. For confocal microscopy, cardiomyocytes were plated on laminin-coated glass coverslips. Free fatty acids (FA) consisted of a 1:1 mix of palmitate (C16:0) and oleate (C18:1 n-9) bound to bovine serum albumin (BSA), at a final total concentration of 0.4 mM FA; TPA or AICAR were added at the time of plating. Small molecules GPAT and DGAT inhibitors were also added at the time of plating. The GPAT inhibitor FSG67^13^ was used at a final concentration of 30 µM, the DGAT inhibitor A922500 at a final concentration of 1 µM and the DGAT inhibitor T863 at a final concentration of 100 nM^14,15^. The culture medium was renewed every 2–3 days, and subsequent analyses were performed on day 7. At this time point, control cardiomyocytes display a well-differentiated phenotype with stable insulin responsiveness^43^.

### Confocal microscopy

For confocal fluorescent microscopy, cardiomyocytes cultured on laminin-coated glass coverslips were washed with ice-cold PBS and fixed with 4 mM paraformaldehyde in PBS for 20 min at room temperature. Fixation was quenched with 200 mM glycine in PBS and cardiomyocytes permeabilized with 0.3% Triton X-100 in PBS for 3 min. Non-specific dye or antibody binding was reduced by preincubation with 3% BSA and 0.1% Tween-20 in PBS. Neutral lipids were stained with 2.5 μg/ml Bodipy 493/503 for 30 min^47^; F-actin counterstaining was obtained with AlexaFluor 543-labeled phalloidin (2 U/ml). Following washes with PBS and H_2_O, coverslips were mounted on glass slides with ProLong Diamond antifade containing DAPI for DNA staining. Cardiomyocytes were examined with a Zeiss LSM800 confocal microscope, using a × 63 oil immersion objective. One-micrometer-thick confocal slices were acquired throughout the thickness of the cardiomyocytes and Z-stack projections obtained with the ImageJ software in maximum intensity projection mode. Image luminosity and contrast were digitally enhanced, taking care to apply the same linear adjustments to images from different experimental groups^48^.

### Glucose uptake measurement

Glucose transport was estimated by measuring 2-deoxyglucose (2-DG) uptake, as previously described^43,49^. Briefly, cardiomyocytes were incubated in M199 containing 10 nM [2,6-^3^H]-2-DG (ANAWA Clinisciences Group; 1–2 μCi/ml) and 5.5 mM cold glucose at 37 °C for 1 h, in the presence or absence of glucose transport agonists. Glucose transport agonists used were insulin (10^−6^ M) or oligomycin (10^−6^ M), a mitochondrial F_O_-ATPase inhibitor, to induce metabolic stress. 2-DG uptake was halted by three washes with ice-cold PBS before lysis in 1 ml 0.1 M NaOH. Two 20-μl aliquots were taken for protein content determination and the remaining NaOH lysate assayed for radioactivity in a TriCARB 1900 TR liquid scintillation analyzer (Packard).

### Immunoblot analysis

Following stimulation with insulin or oligomycin, incubations were terminated by three washes in ice-cold PBS before solubilizing cells in 200 μl lysis buffer containing 150 mM NaCl, 50 mM Tris–HCl (pH 7.5), 1 mM EDTA, 0.5% sodium deoxycholate, 1% Igepal CA 630, Halt protease, and phosphatase inhibitor Cocktail (Pierce, Thermo Scientific). Proteins (30 μg) from each sample were separated on SDS-PAGE gels and transferred onto polyvinylidene difluoride membranes. Primary and secondary antibodies used for western blot analysis are listed in Supplemental Table. For all blots, incubation with the primary antibody was overnight at 4 °C, incubation with the secondary antibody for 1 h at room temperature. Densitometric analysis of chemiluminescent signals captured with a LAS-4000 Luminescent Image Analyzer (Fujifilm) was performed using the ImageJ software (National Institutes of Health, http://rsb.info.nih.gov/ij). For each separate membrane, the data were normalized such that the highest intensity, regardless of treatment, was equal to one.

### Preparation of cytosol and membrane fractions

Cytosol and membrane fractions of cultured cardiomyocytes were obtained as previously described^50^. Cardiomyocytes were washed in ice-cold PBS and scraped in buffer A containing 20 mM Tris–HCl pH 7.5, 2.5 mM EGTA, 1 mM EDTA, 100 mM NaF, 2 mM dithiothreitol, and Halt protease and phosphatase inhibitor cocktail. The suspension was sonicated with four 5-s bursts on ice and then centrifuged at 1500 × g for 10 min. The supernatant was then ultracentrifuged at 100,000 × g for 45 min at 4 °C. The pellet containing the membrane fraction was solubilized in buffer A containing 1% Triton X-100, sonicated, and centrifuged at 15,000 × g for 15 min at 4 °C to retain the supernatant.

### Lipidomics analysis

Cardiomyocytes were washed three times in ice-cold PBS without Ca^2+^ and Mg^2+^ and detached with trypsin/EDTA for 5 min. Trypsin activity was stopped by M199 medium + 20% fetal calf serum and the suspension was centrifugated for 1 min at 500 rpm. The pellet was washed three times in ice-cold PBS without Ca^2+^ and Mg^2^, resuspended in ice-cold PBS without Ca^2+^ and Mg^2^ and sonicated. Shotgun lipidomics analysis of the samples was outsourced to Lipotype GmbH (www.lipotype.com; Dresden, Germany).

Lipids were extracted using chloroform and methanol^51^. Samples were spiked with lipid class-specific internal standards prior to extraction. After drying and re-suspending in mass spectrometry (MS) acquisition mixture, lipid extracts were subjected to mass spectrometric analysis. Mass spectra were acquired on a hybrid quadrupole/Orbitrap mass spectrometer (Q Exactive Plus; Thermo Scientific) equipped with an automated nano-flow electrospray ion source in both positive and negative ion mode. Lipid identification using LipotypeXplorer^52^ was performed on unprocessed (*.raw format) mass spectra. For MS-only mode, lipid identification was based on the molecular masses of the intact molecules. MS/MS mode included the collision-induced fragmentation of lipid molecules and lipid identification was based on both the intact masses and the masses of the fragments. Prior to normalization and further statistical analysis, lipid identifications were filtered according to mass accuracy, occupation threshold, noise and background. Lists of identified lipids and their intensities were stored in a database optimized for the particular structure inherent to lipidomic datasets. The intensity of lipid class-specific internal standards was used for lipid quantification.

### High-performance thin-layer chromatography (HPTLC)

High-Performance Thin-Layer Chromatography of neutral lipids was performed as recently described^53^. Briefly, lipids from cell pellets were extracted using the protocol of Bligh and Dyer^54^ and spotted on HPTLC Silica Gel60 plates (Merck, 1.05631.0001) using an automated sampler (CAMAG ATS 4). The HPTLC plate was pre-washed in chloroform:methanol (1:1) and dried in a vacuum chamber.

For neutral lipids separation, lipid standards (cholesterol, cholesteryl ester, sphingomyelin, phosphatidylcholine, phosphatidylethanolamine and triacylglycerol) and cell lipid extracts resuspended in chloroform:methanol:water 20:9:1 were spotted on the plate using the automated system. The plate was developed first in chloroform:methanol:ammonium hydroxide (65:25:4) for 5 cm, dried briefly and further developed in hexane:diethyl ether:acetic acid (80:20:2) for 9 cm.

After separation, the HPTLC plate was dried in a vacuum chamber for 30 min. Lipids were visualized using a method adapted from Churchward^55^. Briefly, a CuSO_4_ staining solution was prepared: 5 g of CuSO_4_ dissolved in 40 ml water, filtered, mixed with 4.7 ml of 85% ortho-phosphoric acid and filled up to 50 ml with water. 10 ml of freshly prepared staining solution was poured on the TLC plate, incubated for 1 min, decanted and the plate was dried in the vacuum chamber for 15 min. Lipids were then charred at 145 °C for 7.5 min. The plate was visualized in fluorescence light at 488 nm (ChemiDoc MP, Bio-Rad). Lipid spots were quantified from the fluorescent-inverted pictures using densitometry with the ImageJ software.

### Fatty acid uptake and oxidation measurements

Palmitate oxidation was estimated based on the rate of transfer of ^3^H from [9,10-^3^H]palmitate to ^3^H_2_O^56^. Cardiomyocytes were incubated for 60 min in medium containing palmitate (0.05 mM), oleate (0.05 mM), and 1 μCi/ml [9,10-^3^H]palmitate (ANAWA Clinisciences Group) complexed to bovine serum albumin (0.2 mM). At the end of the incubation period, the incubation medium (1 ml) was retrieved, immediately mixed with 1 ml of ice-cold 10% trichloracetic acid and centrifuged at 2,200 g for 10 min at 4 °C. The supernatant was neutralized with 250 µl of NaOH 6 M. ^3^H_2_O in the supernatant was separated from [9,10-^3^H]palmitate by anion exchange chromatography in Dowex 1 × 4. ^3^H_2_O eluted from the Dowex column was counted by liquid scintillation.

Cardiomyocytes were washed twice with ice-cold PBS, dissolved in 0.1 M NaOH. Twenty-µl aliquots were taken for protein content determination and the remaining NaOH lysate assayed for radioactivity in a TriCARB 1900 TR liquid scintillation analyzer (Packard). Palmitate uptake was estimated from the sum of ^3^H label transferred to ^3^H_2_O and ^3^H label remaining in the cardiomyocytes.

### Statistics

Data are presented as mean ± SEM obtained from replicated experiments. Data were compared by one-way or two-way ANOVA (Prism 7, GraphPad Software) followed by post hoc testing for false discovery rates by the method of Benjamini and Yekutieli^57^. Post hoc testing indicated a positive discovery when the false discovery rate q was < 0.05. Throughout the article, the following symbols are used for positive discoveries (q values): * indicates a significant effect of insulin or oligomycin stimulation as compared with unstimulated cardiomyocytes having received the same chronic treatment; # indicates a significant effect of chronic FA exposure, as compared with cardiomyocytes not exposed to FA undergoing the same acute stimulation; § indicates a significant effect of chronic TPA or AICAR exposure as compared with cardiomyocytes not exposed to TPA or AICAR undergoing the same acute stimulation; † indicates a significant effect of chronic DGAT or GPAT inhibition (but with the same exposure—or absence thereof—to FA, TPA or AICAR), as compared with cardiomyocytes not exposed to DGAT or GPAT inhibitor undergoing the same acute stimulation.

### Supplementary Information

Supplementary Information 1.

Supplementary Figure S1.

Supplementary Figure S2.

Supplementary Figure S3.

Supplementary Figure S4.

Supplementary Information 6.

Supplementary Information 7.

## Data Availability

The complete lipidomics data obtained are available as supplemental data to this study. Other datasets generated during and/or analyzed during the current study are available from the corresponding author on reasonable request.
