# Supplementary figures and images for: Differential intracellular management of fatty acids impacts on metabolic stress-stimulated glucose uptake in cardiomyocytes
Source: Sci Rep. 2023 Sep 8;13:14805. doi: 10.1038/s41598-023-42072-7 (PMC10491837; doi:10.1038/s41598-023-42072-7)

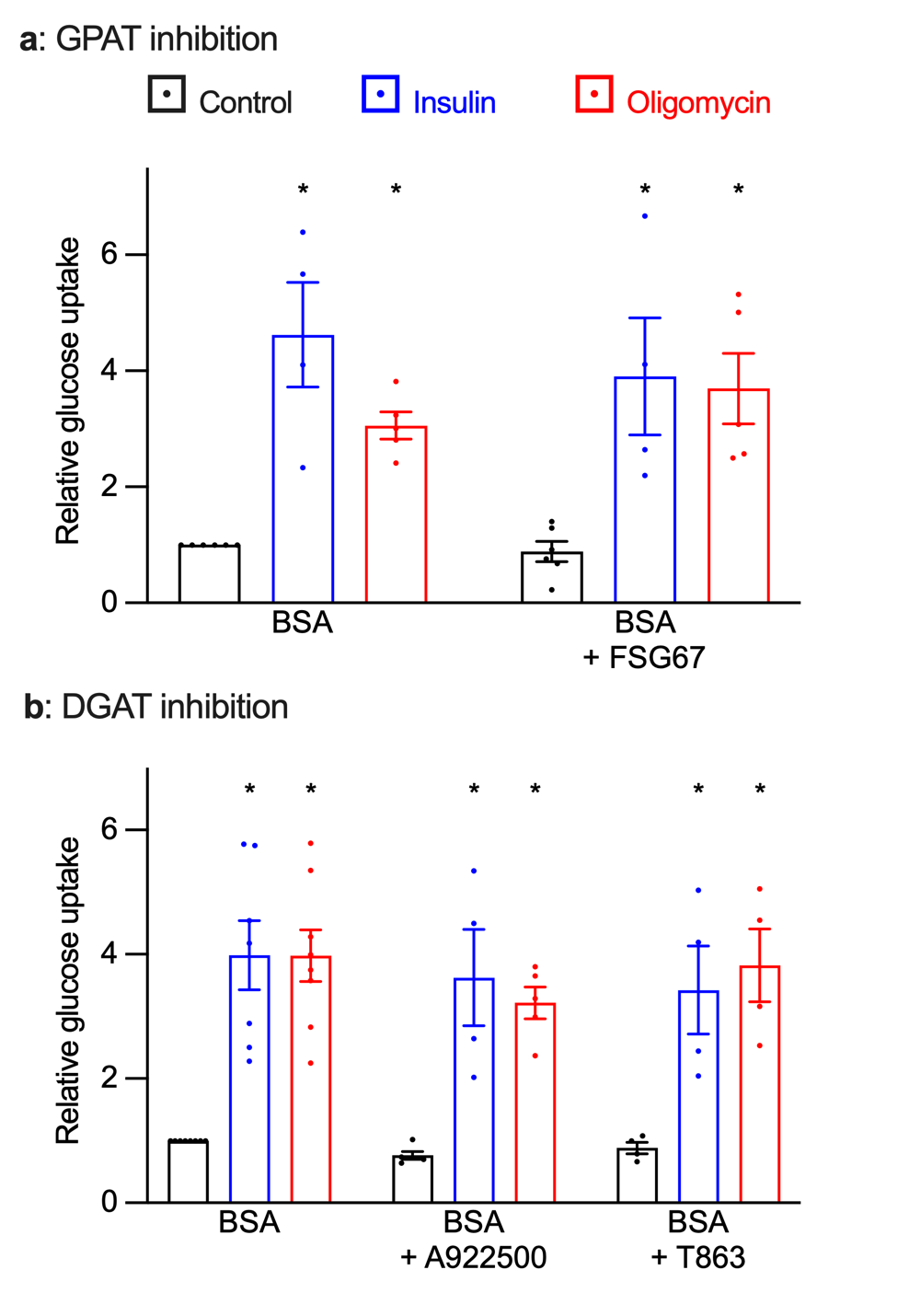

Supplement: Supplementary file 2 — Supplementary Figure S1. [file 41598_2023_42072_MOESM2_ESM.tiff]

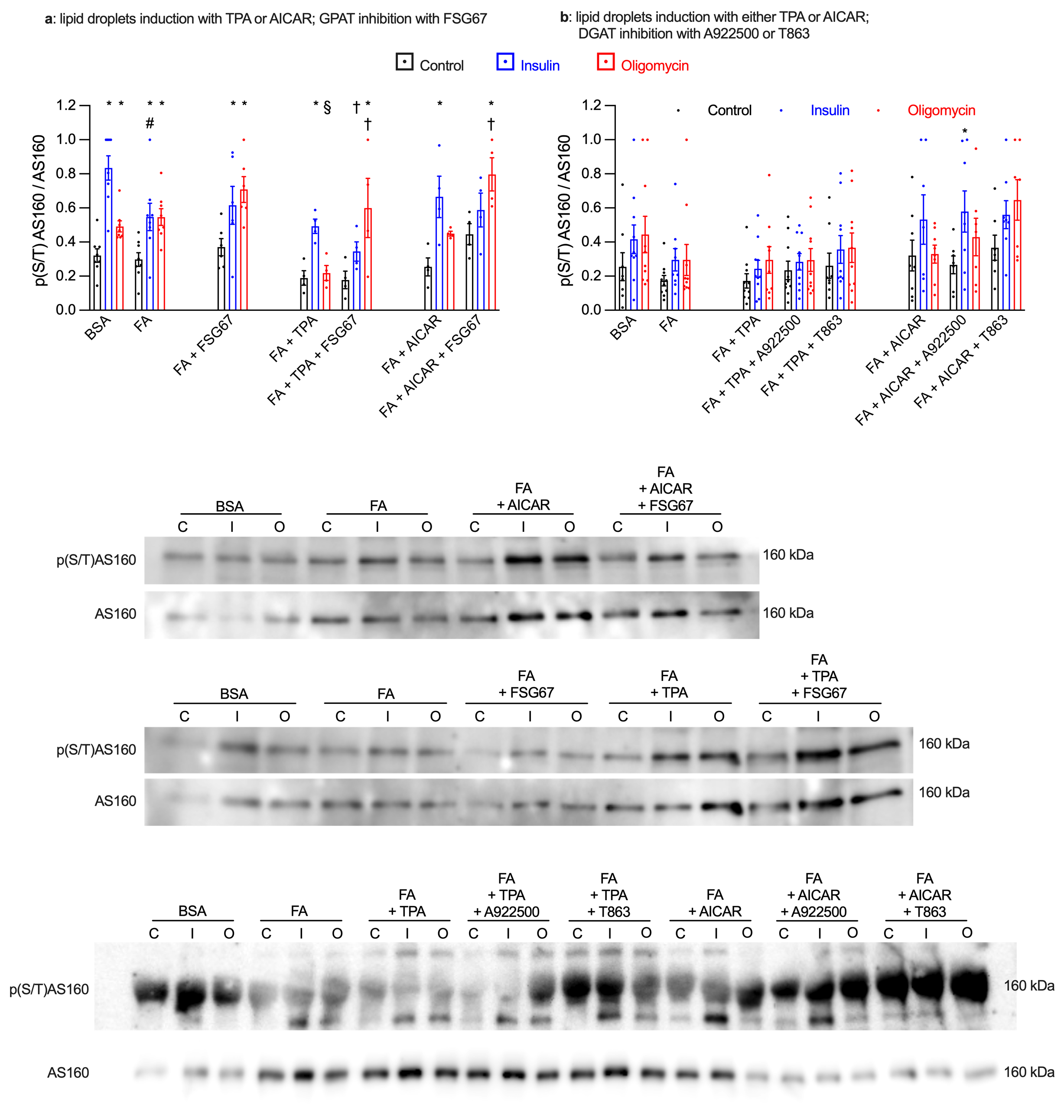

Supplement: Supplementary file 3 — Supplementary Figure S2. [file 41598_2023_42072_MOESM3_ESM.tiff]

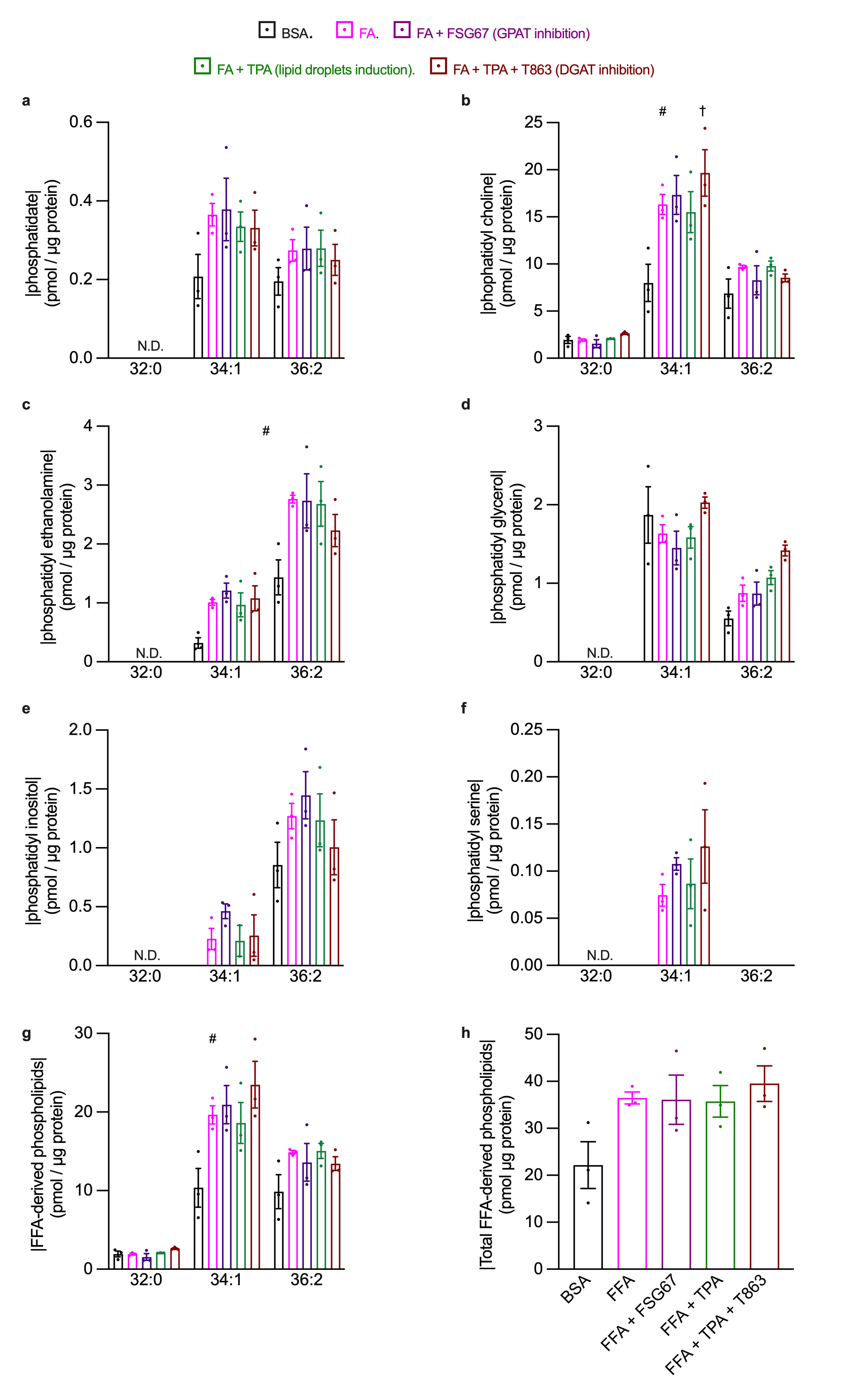

Supplement: Supplementary file 4 — Supplementary Figure S3. [file 41598_2023_42072_MOESM4_ESM.tiff]

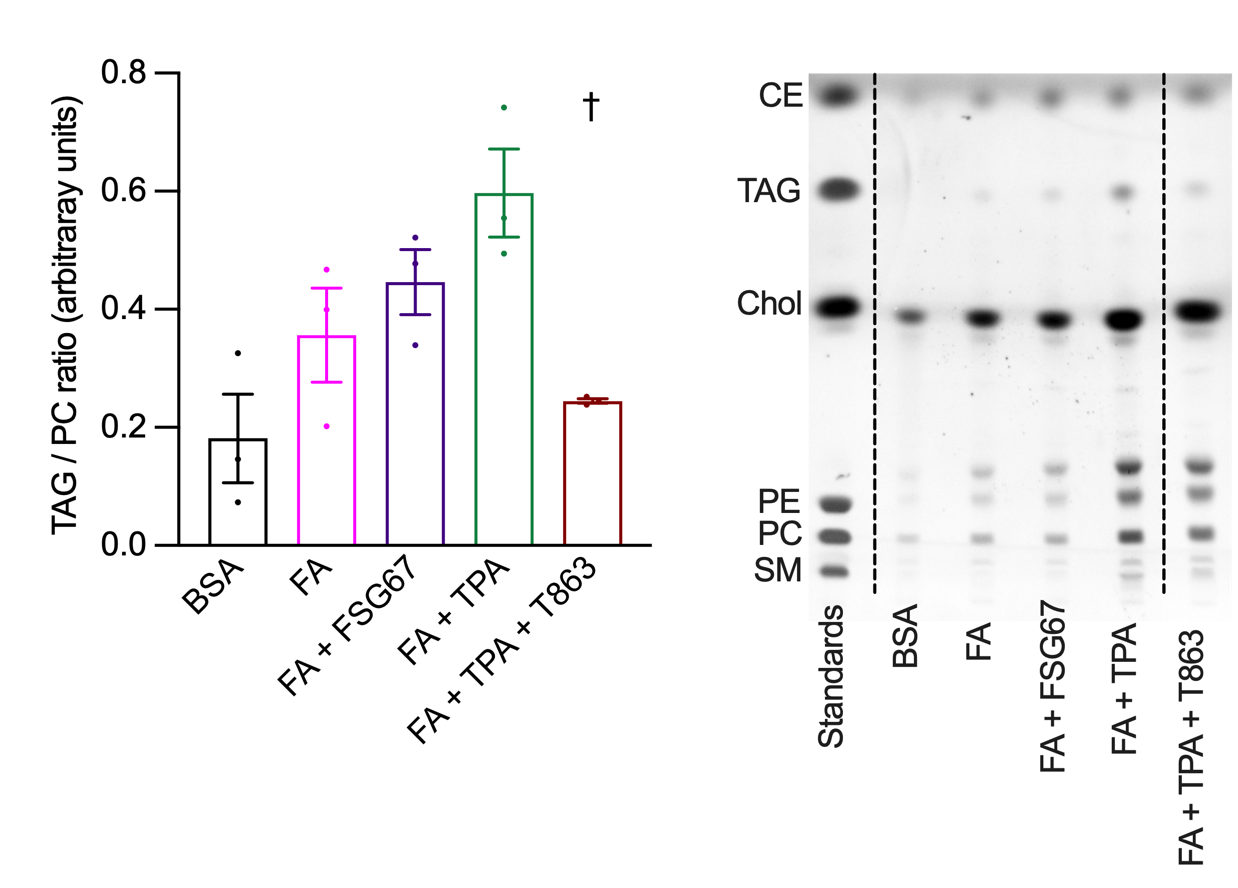

Supplement: Supplementary file 5 — Supplementary Figure S4. [file 41598_2023_42072_MOESM5_ESM.tiff]
