# Supplementary material for: Differential intracellular management of fatty acids impacts on metabolic stress-stimulated glucose uptake in cardiomyocytes
Source: Sci Rep. 2023 Sep 8;13:14805. doi: 10.1038/s41598-023-42072-7 (PMC10491837; doi:10.1038/s41598-023-42072-7)
Supplement: Supplementary file 6 — Supplementary Information 6. [file 41598_2023_42072_MOESM6_ESM.docx]

**Differential intracellular management of fatty acids impacts on metabolic stress-stimulated glucose uptake in cardiomyocytes.**

Ettore Vanni^1^, Karina Lindner^2^, Anne-Claude Gavin^2^ & Christophe Montessuit^1*^

# Supplemental material

**Supplemental Figure 1. Neither DGAT nor GPAT inhibitors affect glucose uptake in cardiomyocytes cultured in absence of lipids** Primary cardiomyocytes were cultured for 7 days with fatty acid-free BSA and the GPAT inhibitor FSG67 (30 µM; panel **a**) or the DGAT inhibitors A922500 (1 µM) or T863 (100 nM; panel **b**) or dimethyl sulfoxide as the vehicle control. Glucose uptake was then measured during 1 hour exposure to either 1 µM insulin (blue bars), 1 µM oligomycin (red bars) or control (black bars). Results are shown as mean ± SEM; n = 4-8. *: significant effect (q < 0.05) of insulin or oligomycin.

**Supplemental Figure 2. Variations in AS160 phosphorylation match variations in AMPKα phosphorylation** Primary cardiomyocytes were cultured for 7 days with or without 0.4 mM fatty acids (FA), 100 nM TPA or 0.2 mM AICAR. Additional treatments were the GPAT inhibitor FSG67 (30 µM) or dimethyl sulfoxide as the vehicle control (panel **a**) or the DGAT inhibitors A922500 (1 µM) or T863 (100 nM) or dimethyl sulfoxide as the vehicle control (panel **b**). Cardiomyocytes were then stimulated for 10 minutes with 1 µM insulin, for 20 minutes with 1 µM oligomycin or left unstimulated. Cells were extracted and submitted to western blot analysis the measure the expressions of phosphorylated (S/T residues)) and total AS160. The ratios of phosphorylated/total AS160 were than calculated and are displayed in the graphs. Results are shown as mean ± SEM; n = 4-10. *: significant effect (q < 0.05) of insulin or oligomycin; #: significant effect of FA; §: significant effect of TPA; †: significant effect of the GPAT or the DGAT inhibitor. Bottom panels show representative western blots.

**Supplemental Figure 3. FA-derived cellular phospholipids concentrations** Primary cardiomyocytes were cultured for 7 days with or without 0.4 mM FA, 100 nM TPA and the GPAT inhibitor FSG67, the DGAT inhibitor T863 or DMSO. Total cellular lipids were then extracted and analyzed by mass spectrometry. Results show A-derived cellular phospholipid concentrations detailed by class and species (panels **a-f**), pooled by class (panel **g**) and pooled by class and species (panel **h**). N.D.: Not detected. Results are shown as mean ± SEM; n = 3. #: significant effect of FA; †: significant effect of the DGAT inhibitor.

**Supplemental Figure 4. TAG content analysis by thin-layer chromatography**

Primary cardiomyocytes were cultured for 7 days with or without 0.4 mM fatty acids (FA), 100 nM TPA and the GPAT inhibitor FSG67, the DGAT inhibitor T863 or dimethyl sulfoxide. Total cellular lipids were then extracted and analyzed by thin-layer chromatography. The right panel shows a representative thin-layer chromatography plate; dashed lines indicate where the plate image has been cut and spliced. Other lipid standards abbreviations: CE: cholesteryl esters; Chol: cholesterol; PE: phosphatidylethanolamine; PC: phosphatidylcholine; SM: sphingomyelin.

**Supplemental data: Lipidomics_data.xlsx**

MS Excel file of all features detected in the shotgun mass spectrometry experiment.

**Supplemental data: Uncropped_gels.pdf**

Uncropped gels and TLC plate for Figures 3,5,6 and Supplemental Figures 2 and 4

**Supplemental Table : Primary and secondary antibodies used in immunoblot analysis**

| **Antibody** | **Source** | **Catalog #** | **Dilution** |
| --- | --- | --- | --- |
| Primary antibodies | | | |
| AMPKα | Cell Signaling Technologies | 2532 | 1:1000 |
| Phospho-AMPKα (Thr172) | Cell Signaling Technologies | 2535 | 1:1000 |
| Raptor | Cell Signaling Technologies | 2280 | 1:1000 |
| Phospho-raptor (Ser792) | Cell Signaling technologies | 2083 | 1:1000 |
| Connexin 43 | Cell Signaling Technologies | 3512 | 1:1000 |
| Protein Kinase C δ | BD Transduction Laboratories | 610397 | 0.25 µg / ml |
| Phospho-(Ser/Thr) Akt substrate | Cell Signaling Technologies | 9611 | 1:1000 |
| AS160 | Cell Signaling Technologies | 2670 | 1:1000 |
| Secondary antibodies | | | |
| Anti-rabbit IgG, HRP-linked | Cell Signaling Technology | 7074 | 1:5000 |
| Anti-mouse IgG, HRP-linked | Cell Signaling Technology | 7076 | 1:5000 |
