# Supplementary material for: Differential intracellular management of fatty acids impacts on metabolic stress-stimulated glucose uptake in cardiomyocytes
Source: Sci Rep. 2023 Sep 8;13:14805. doi: 10.1038/s41598-023-42072-7 (PMC10491837; doi:10.1038/s41598-023-42072-7)
Supplement: Supplementary file 7 — Supplementary Information 7. [file 41598_2023_42072_MOESM7_ESM.pdf]

Figure 3a uncropped blots

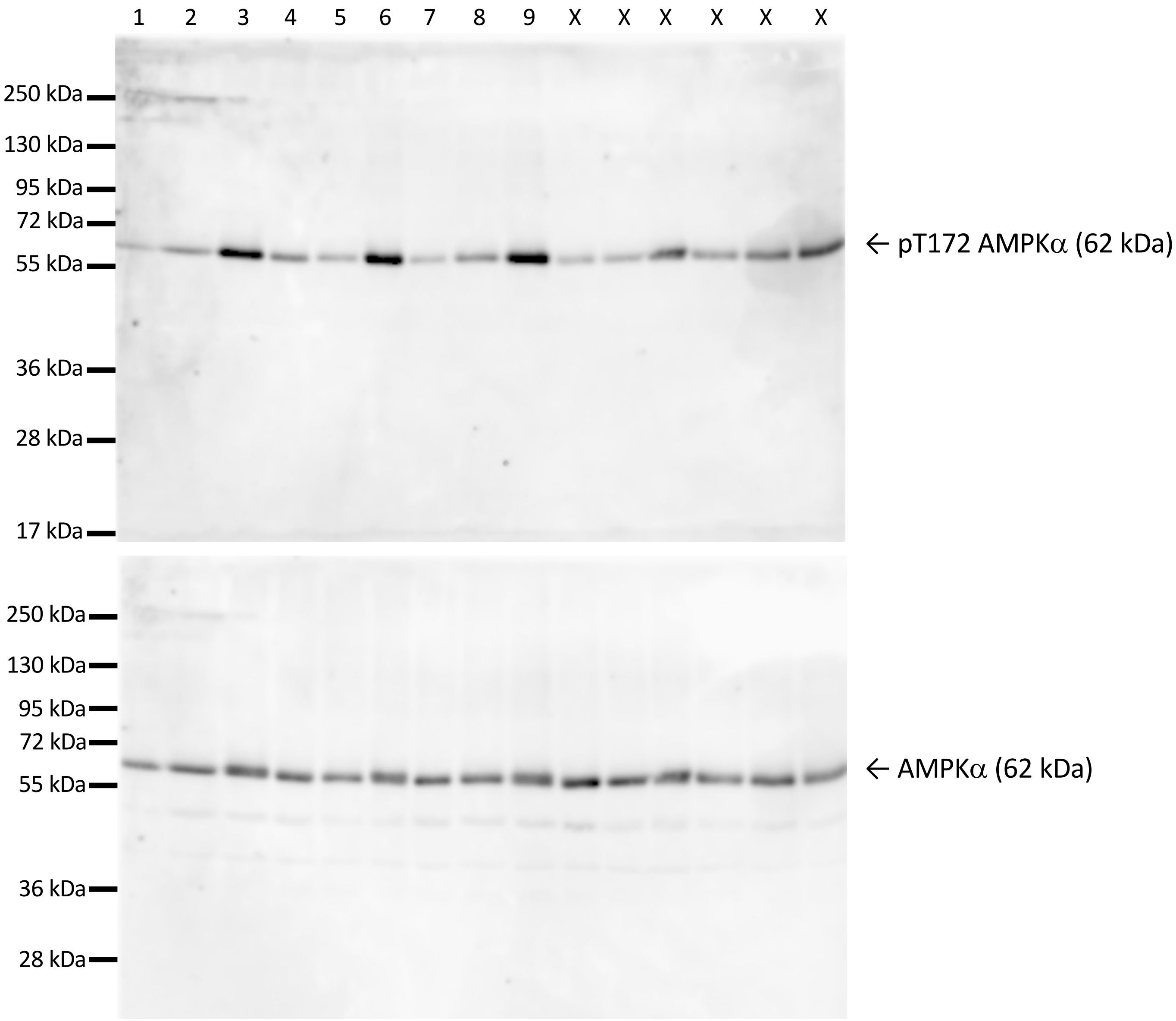

Loading order

Acute stimulation → Chronic treatment ↓	Control	Insulin	Oligomycin
BSA	1	2	3
FA	4	5	6
FA + FSG67	7	8	9

X: unused lanes

Figure 3b uncropped blots

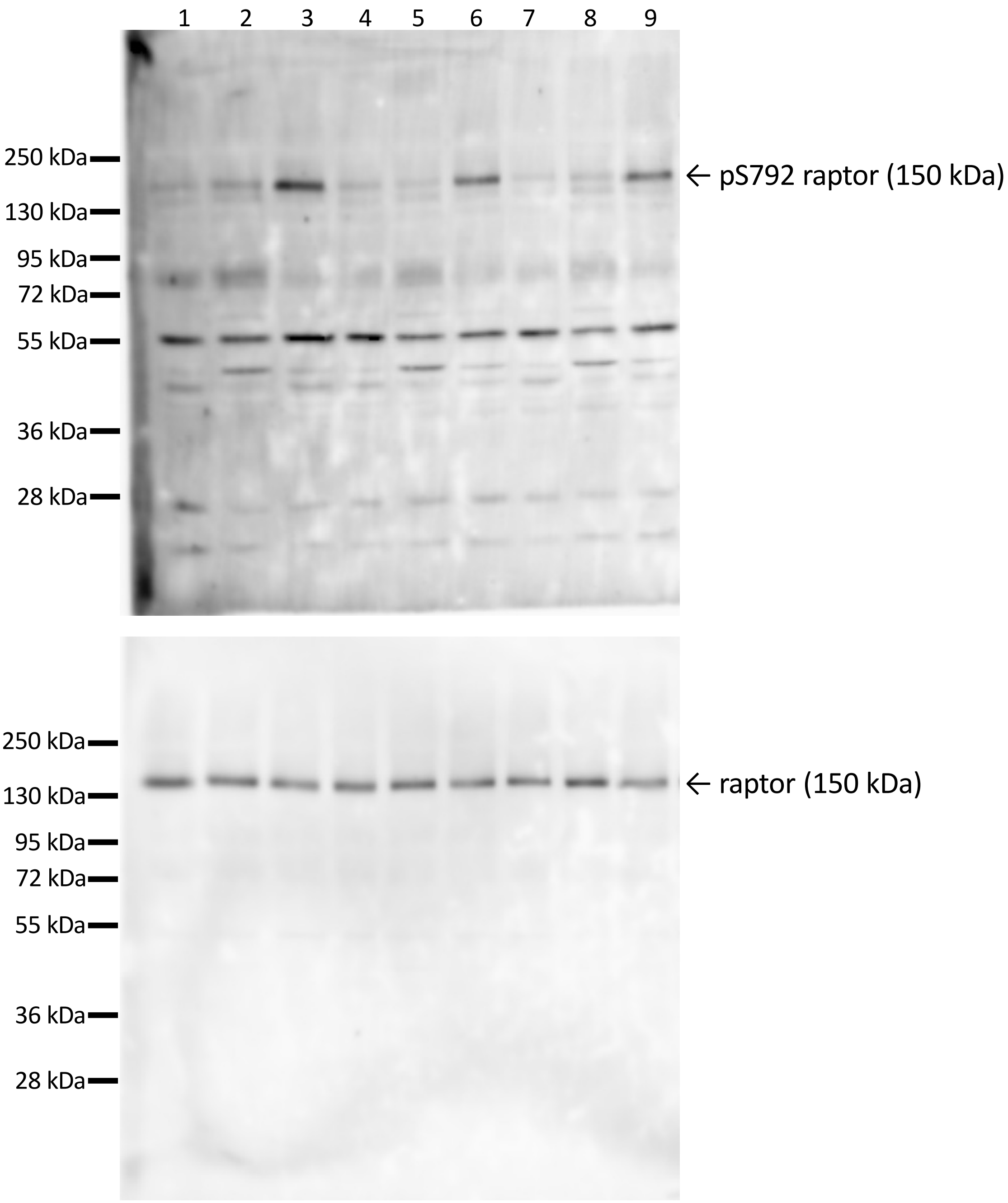

Loading order

Acute stimulation → Chronic treatment ↓	Control	Insulin	Oligomycin
BSA	1	2	3
FA	4	5	6
FA + FSG67	7	8	9

Figure 3c uncropped blots

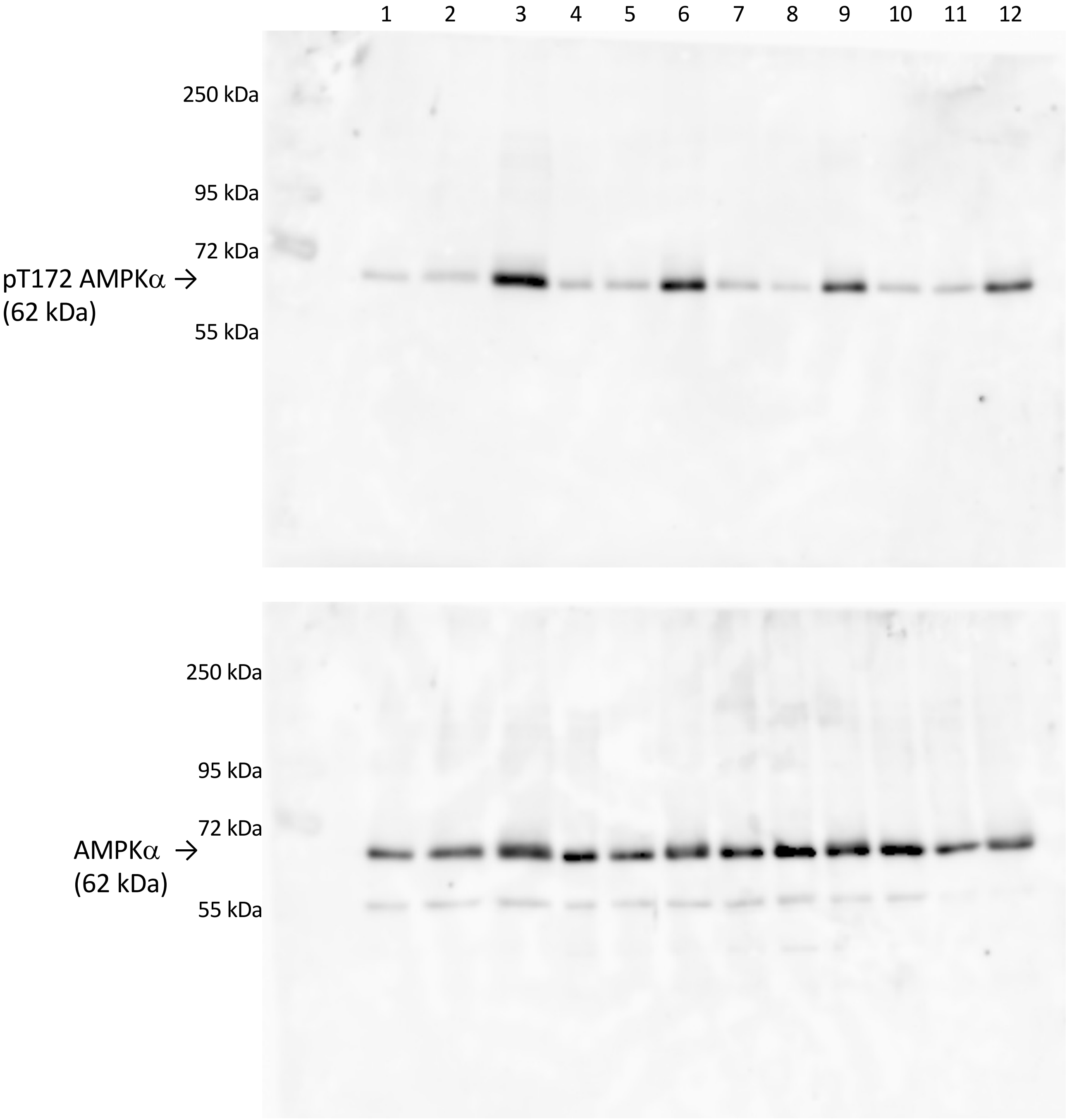

Loading order

Acute stimulation → Chronic treatment ↓	Control	Insulin	Oligomycin
BSA	1	2	3
FA	4	5	6
FA + TPA	7	8	9
FA + TPA + FSG67	10	11	12

Figure 3d uncropped blots

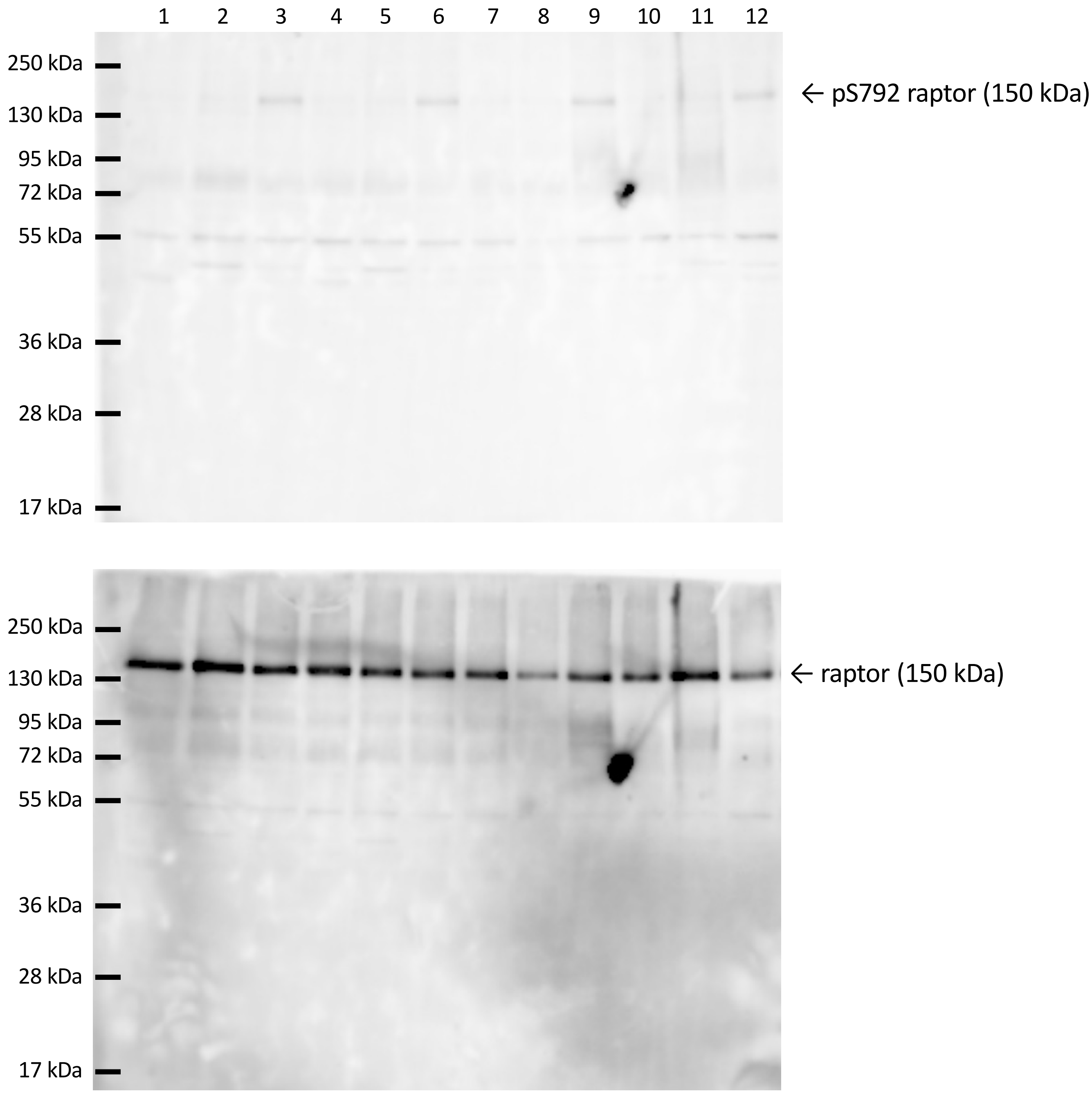

Loading order

Acute stimulation →<br>Chronic treatment ↓	Control	Insulin	Oligomycin
BSA	1	2	3
FA	4	5	6
FA + TPA	7	8	9
FA + TPA + FSG67	10	11	12

Figure 3e uncropped blots (same blots as figure 3a)

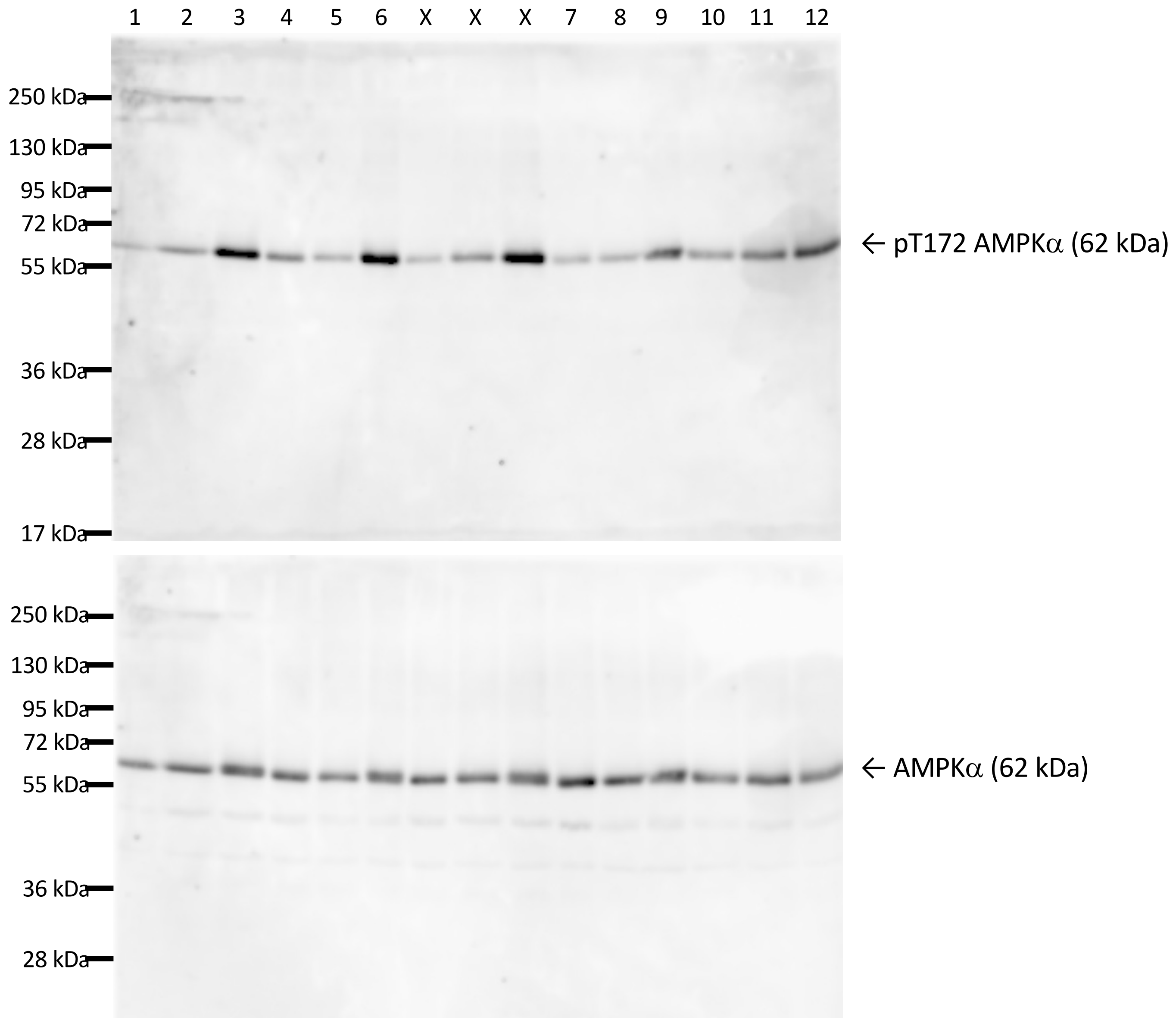

Loading order

Acute stimulation → Chronic treatment ↓	Control	Insulin	Oligomycin
BSA	1	2	3
FA	4	5	6
FA + TPA	7	8	9
FA + TPA + FSG67	10	11	12

X: unused lanes

Figure 3f uncropped blots

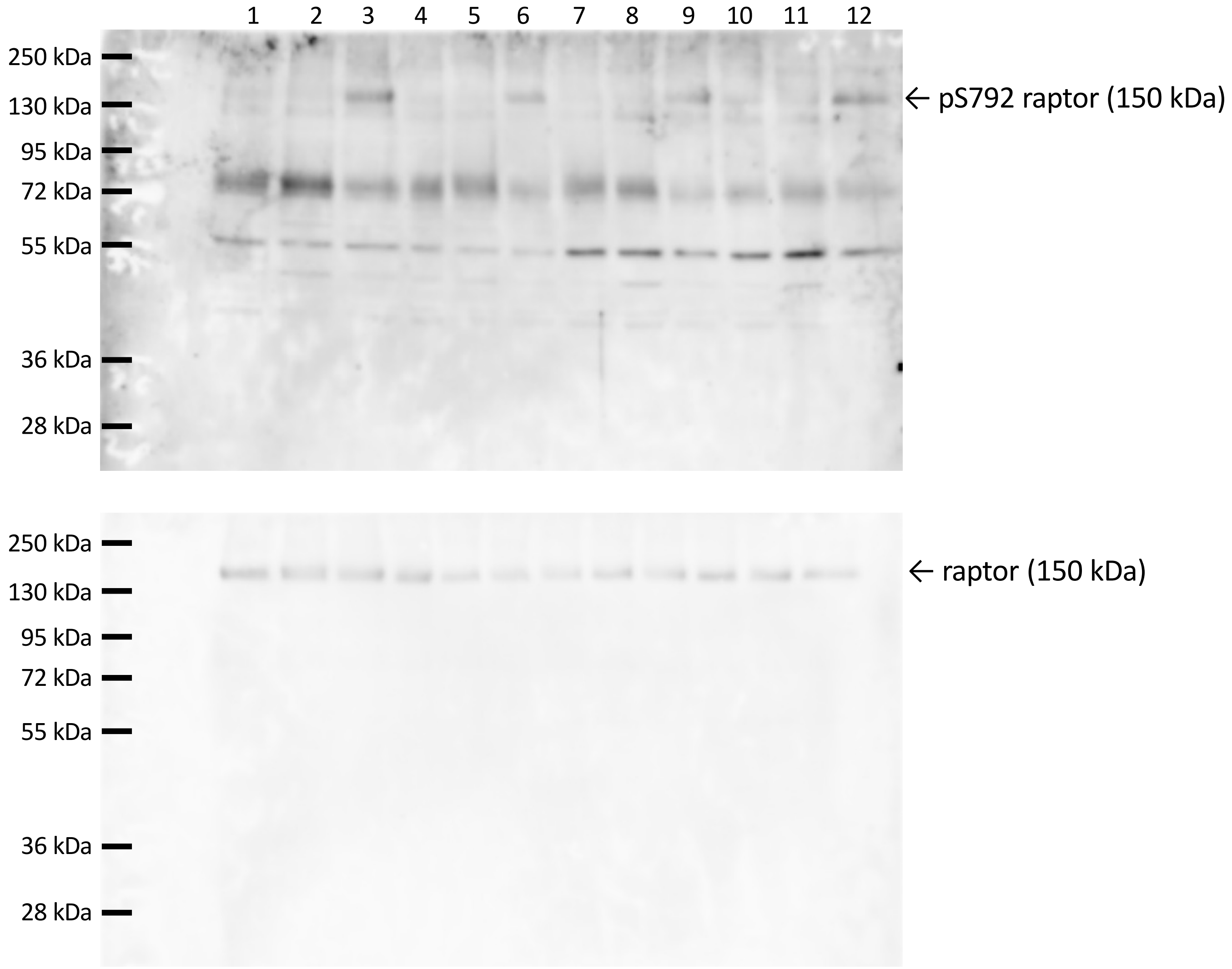

Loading order

Acute stimulation →<br>Chronic treatment ↓	Control	Insulin	Oligomycin
BSA	1	2	3
FFA	4	5	6
FA + AICAR	7	8	9
FA + AICAR + FSG67	10	11	12

<u>Figure 5a and 5c uncropped blots</u>

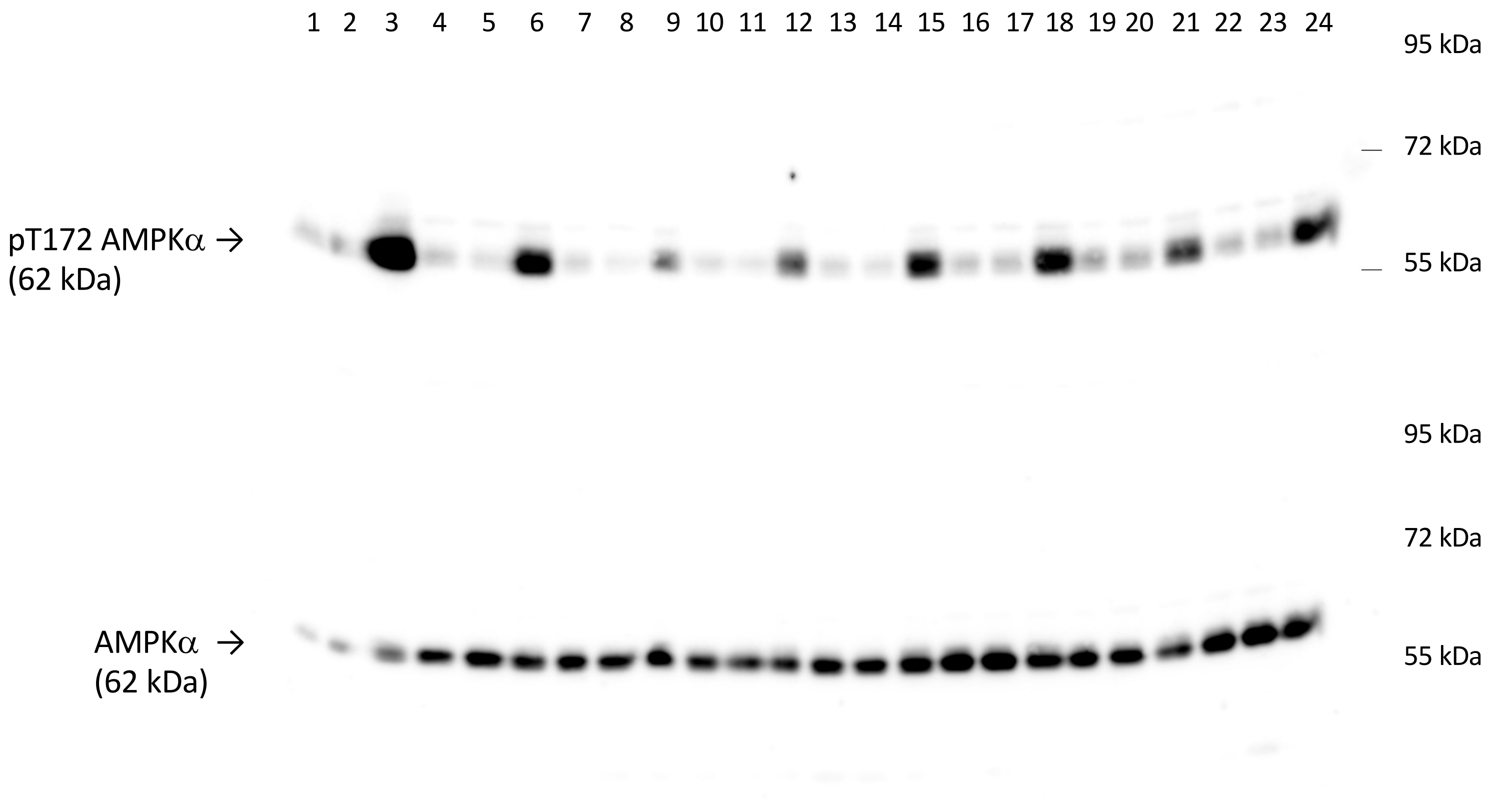

<u>Loading order</u>

Acute stimulation → Chronic treatment ↓	Control	Insulin	Oligomycin
BSA	1	2	3
FA	4	5	6
FA + TPA	7	8	9
FA + TPA + A922500	10	11	12
FA + TPA + T863	13	14	15
FA + AICAR	16	17	18
FA + AICAR + A922500	19	20	21
FA + AICAR + T863	22	23	24

N.B. Despite the high contrast in the pT172 AMPKα and AMPKα blots, all pixels were below saturation.

Figure 5b uncropped blots

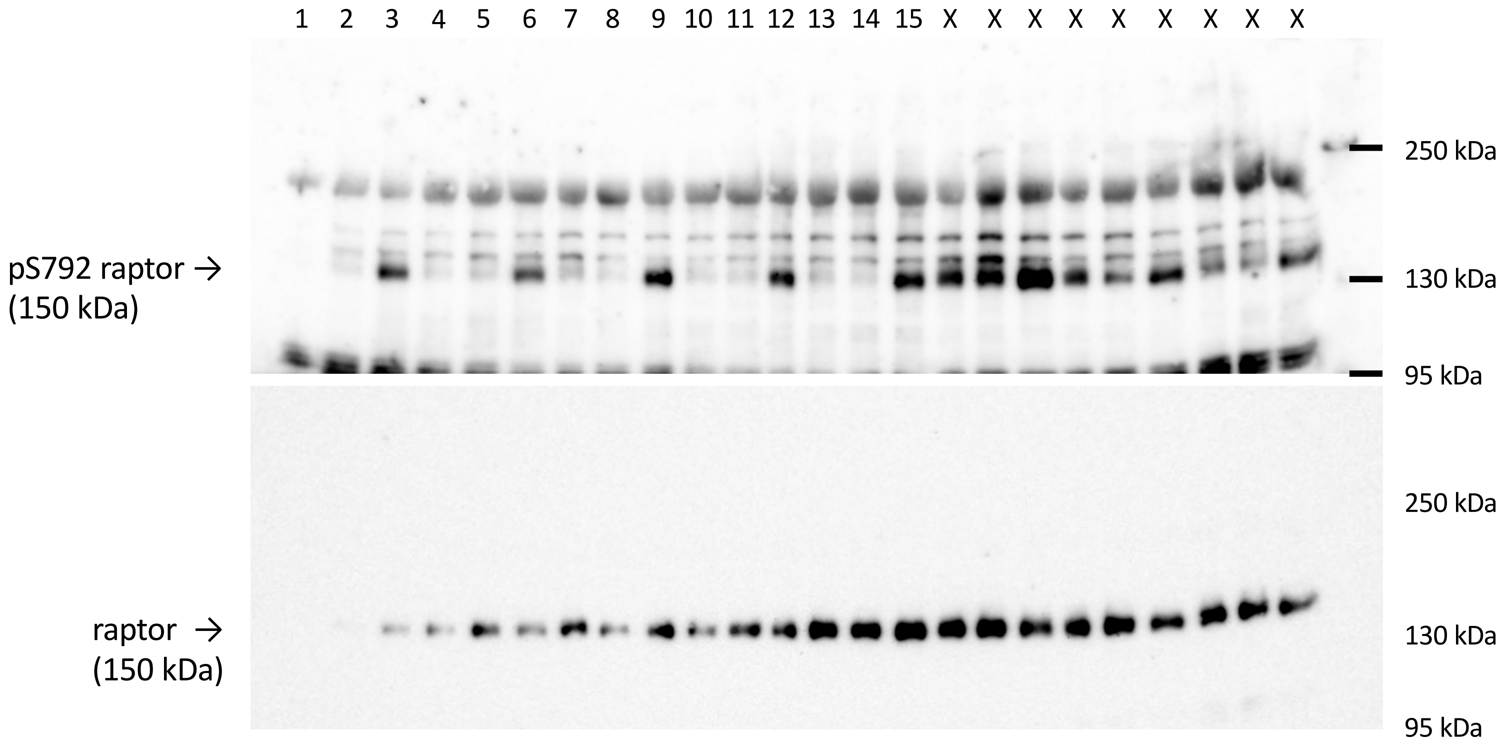

Loading order

Acute stimulation → Chronic treatment ↓	Control	Insulin	Oligomycin
BSA	1	2	3
FA	4	5	6
FA + TPA	7	8	9
FA + TPA + A922500	10	11	12
FA + TPA + T863	13	14	15

X: unused lanes

Figure 5d uncropped blots

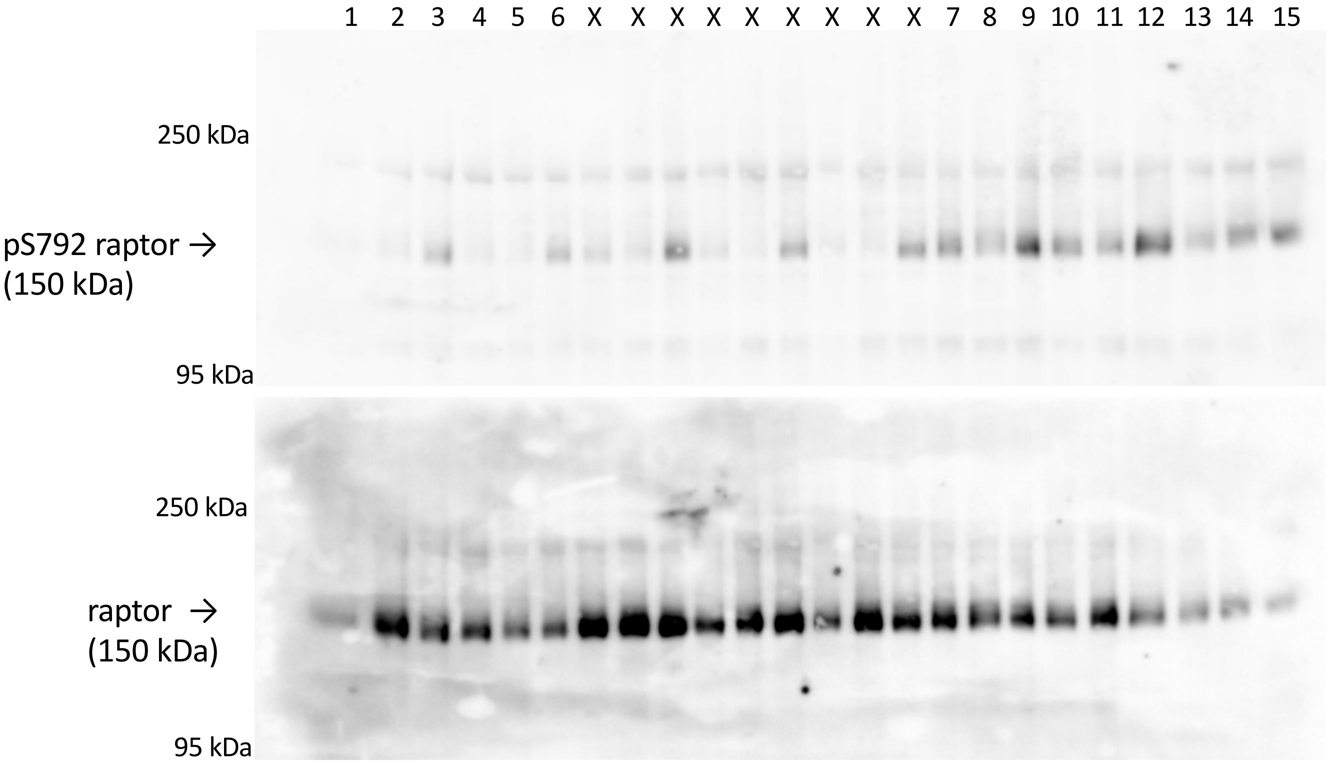

Loading order

Acute stimulation → Chronic treatment ↓	Control	Insulin	Oligomycin
BSA	1	2	3
FA	4	5	6
FA + AICAR	7	8	9
FA + AICAR + A922500	10	11	12
FA + AICAR + T863	13	14	15

X: unused lanes

Figure 6 uncropped PKCδ blots

Uncropped blot for Figure 6; whole cell extract

Uncropped blot for Figure 6; membrane fraction

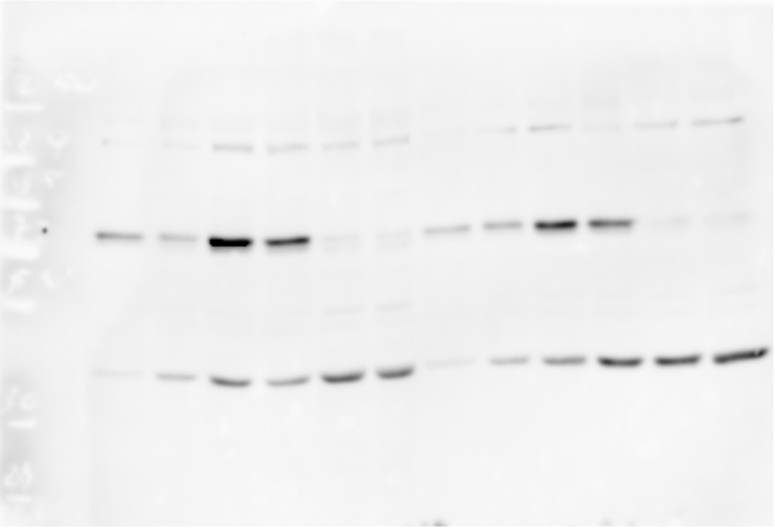

Loading order:

1: BSA
2: FA
3: FA + FSG67
4: FA + TPA
5: FA + TPA +T863
X: unused lanes

Figure 6 uncropped connexin 43 blots

N.B. for these blots the membrane was cut before incubation with the antibody

Uncropped blot for Figure 6; whole cell extract

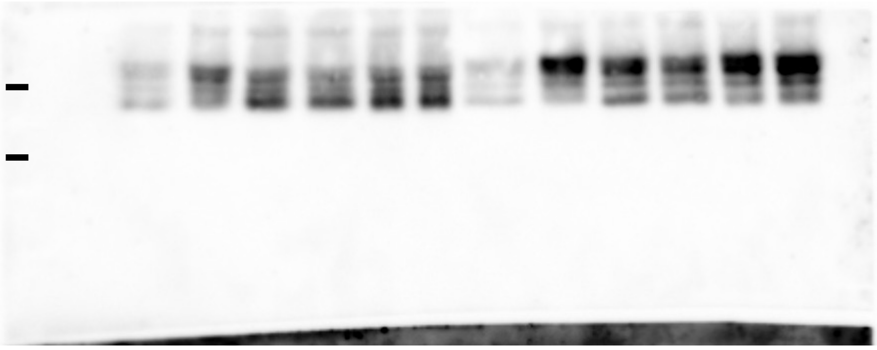

Uncropped blot for Figure 6; membrane fraction

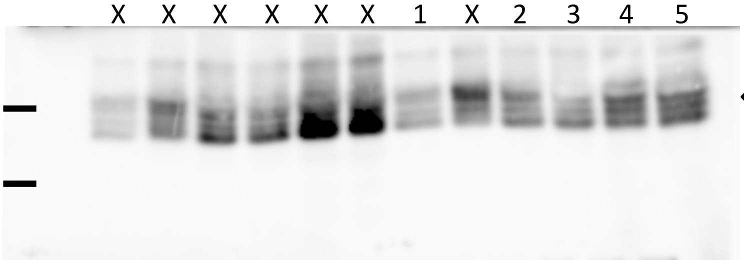

Loading order:

1: BSA
2: FA
3: FA + FSG67
4: FA + TPA
5: FA + TPA +T863
X: unsused lanes

Supplemental Figure 2 uncropped blots 1 & 2

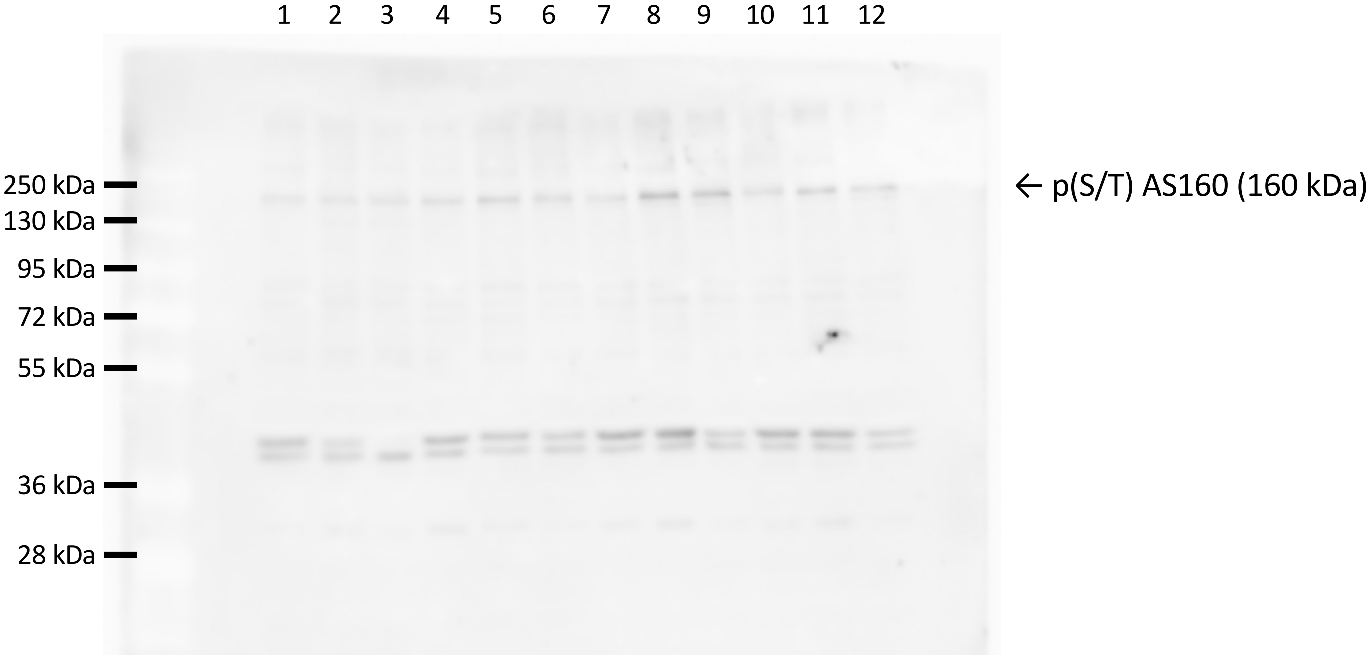

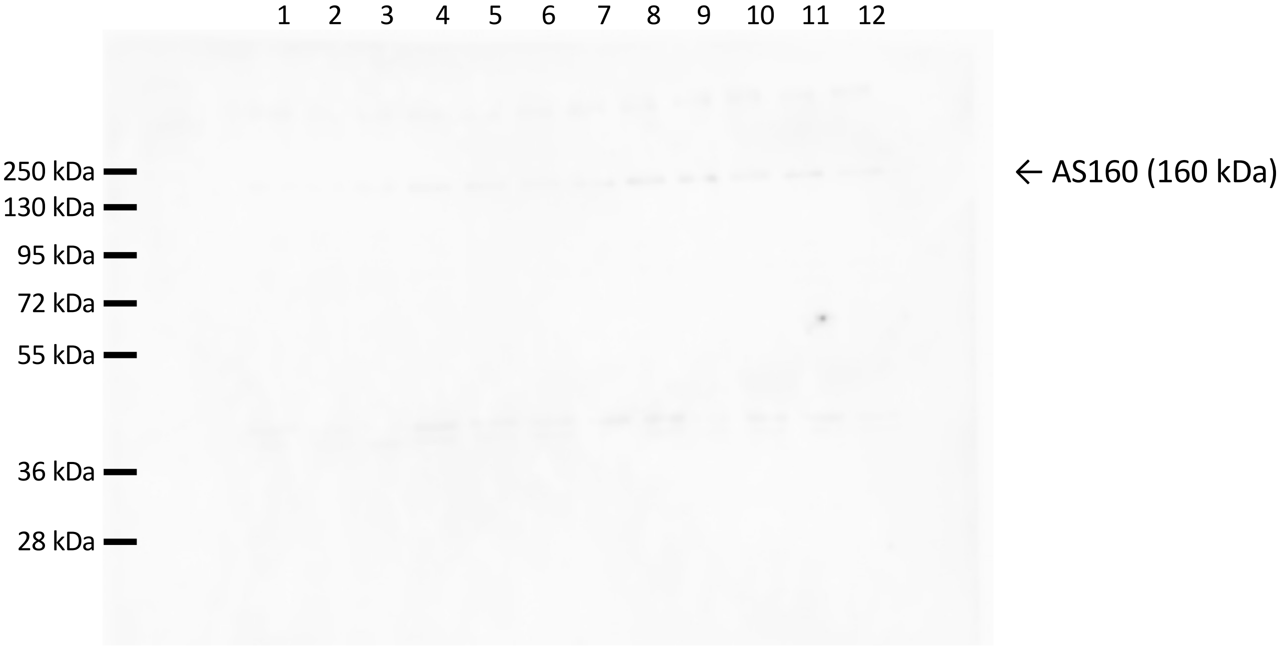

Loading order

Acute stimulation → Chronic treatment ↓	Control	Insulin	Oligomycin
BSA	1	2	3
FA	4	5	6
FA + AICAR	7	8	9
FA + AICAR + FDG67	10	11	12

Supplemental Figure 2 uncropped blots 3 & 4

1 2 3 4 5 6 7 8 9 10 11 12 13 14 15

← p(S/T) AS160 (160 kDa)

1 2 3 4 5 6 7 8 9 10 11 12 13 14 15

← AS160 (160 kDa)

Loading order

Acute stimulation →<br>Chronic treatment ↓	Control	Insulin	Oligomycin
BSA	1	2	3
FA	4	5	6
FA + FSG67	7	8	9
FA + TPA	10	11	12
FA + TPA + FSG67	13	14	15

Supplemental Figure 2 uncropped blots 5 & 6

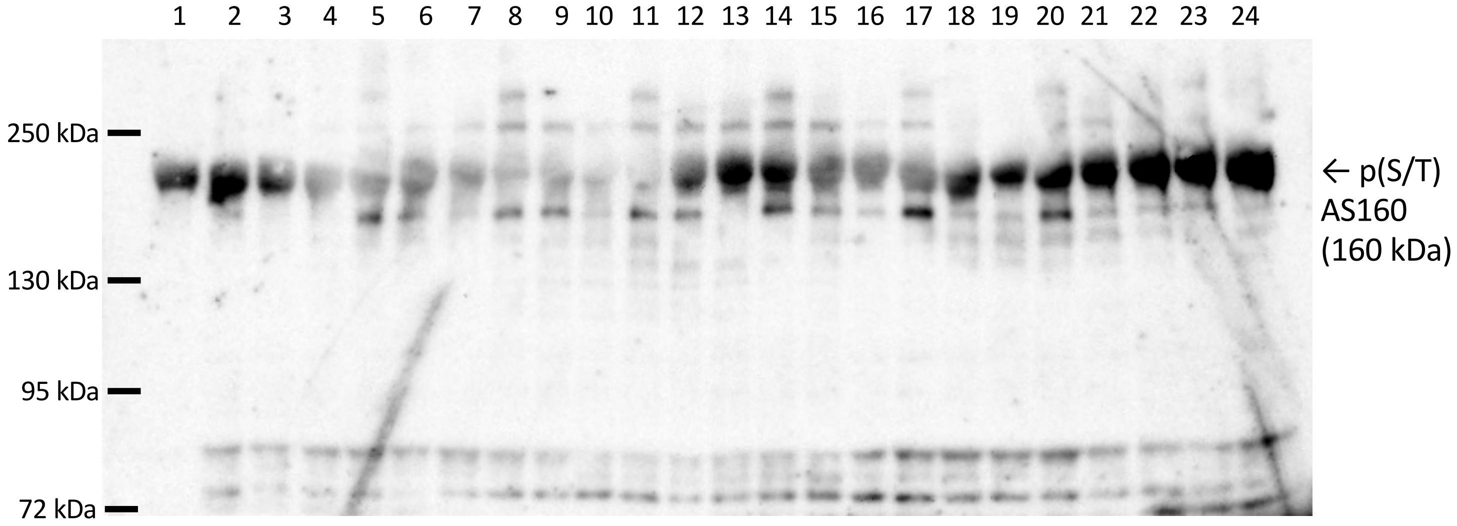

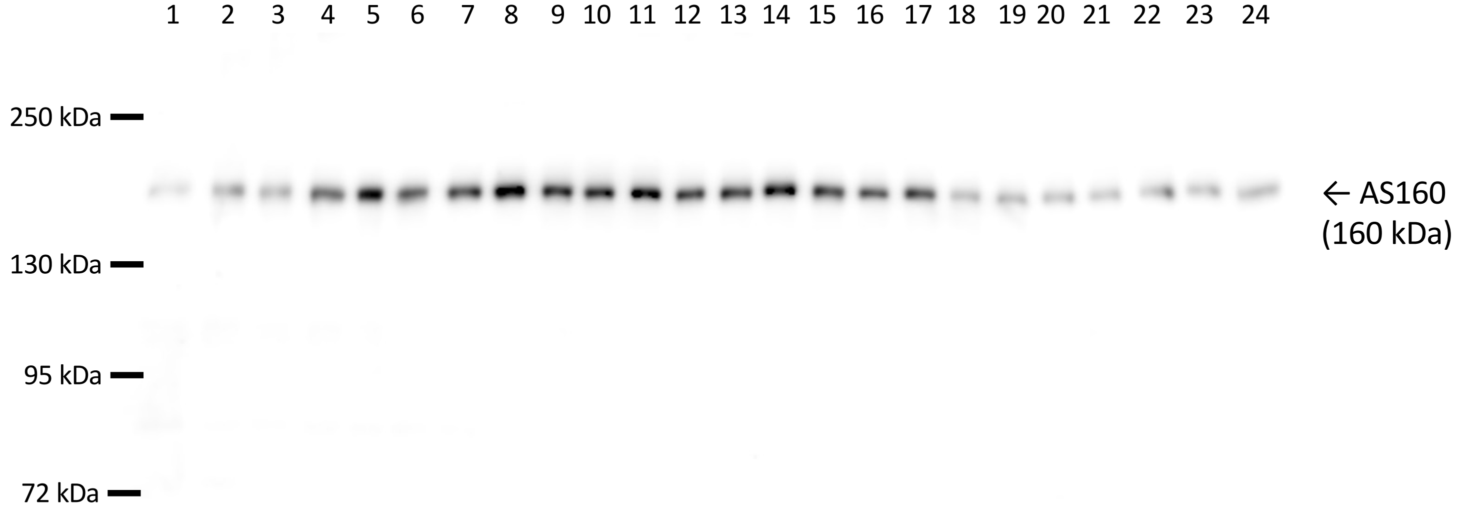

Loading order

Acute stimulation → Chronic treatment ↓	Control	Insulin	Oligomycin
BSA	1	2	3
FA	4	5	6
FA + TPA	7	8	9
FA + TPA + A922500	10	11	12
FA + TPA + T863	13	14	15
FA + AICAR	16	17	18
FA + AICAR + A922500	19	20	21
FA + AICAR + T863	22	23	24

N.B. Despite the high contrast in these blots, all pixels were below saturation.

Supplemental Figure 4 uncropped TLC plate

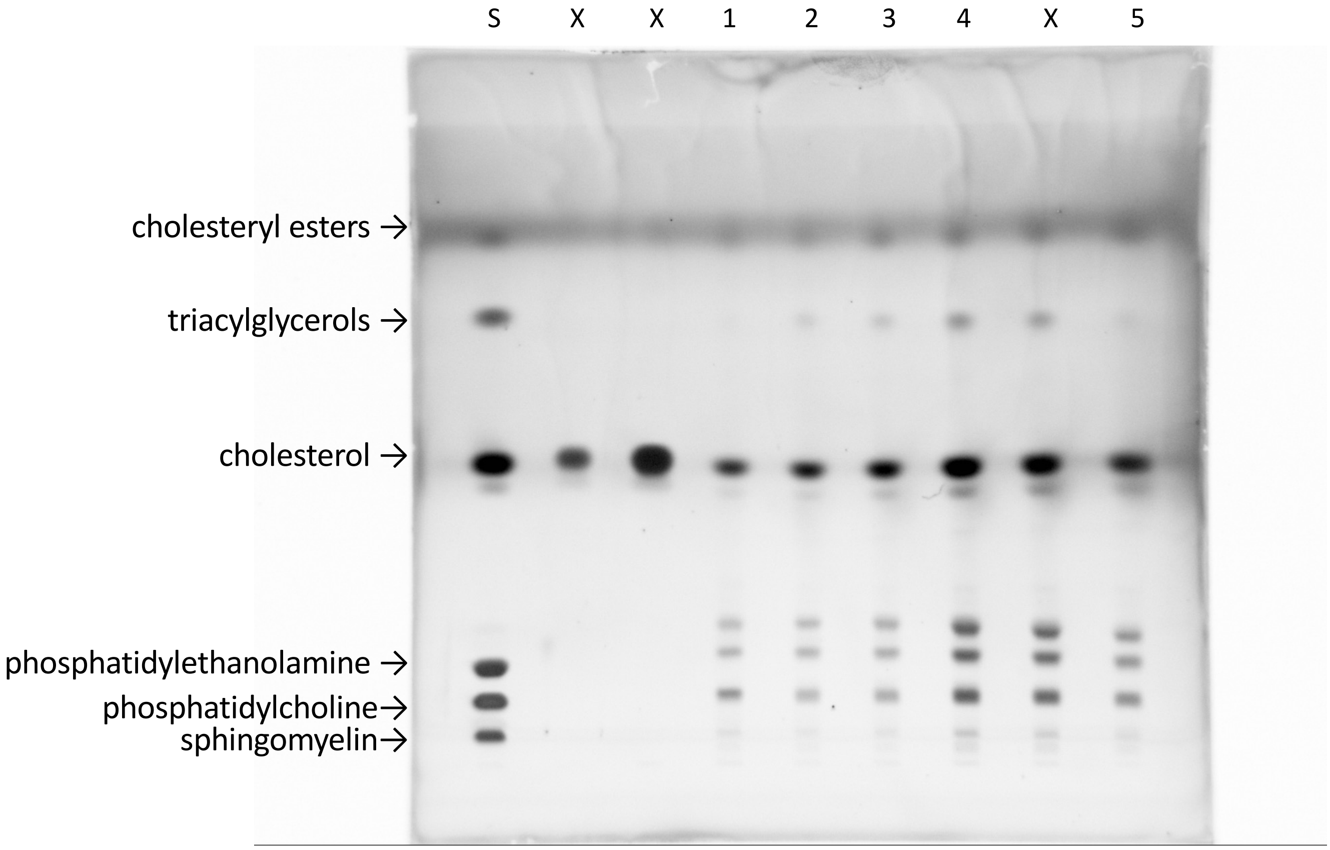

Loading order:

S: lipids standards
1: BSA
2: FA
3: FA + FSG67
4: FA + TPA
5: FA + TPA +T863
X: unused lanes
